# Characterization of the *Paracoccidioides* Hypoxia Response Reveals New Insights into Pathogenesis Mechanisms of This Important Human Pathogenic Fungus

**DOI:** 10.1371/journal.pntd.0004282

**Published:** 2015-12-10

**Authors:** Patrícia de Sousa Lima, Dawoon Chung, Alexandre Melo Bailão, Robert A. Cramer, Célia Maria de Almeida Soares

**Affiliations:** 1 Laboratório de Biologia Molecular, Instituto de Ciências Biológicas, Universidade Federal de Goiás, Goiânia, Goiás, Brazil; 2 Department of Microbiology and Immunology, Geisel School of Medicine at Dartmouth, Hanover, New Hampshire, United States of America; University of California San Diego School of Medicine, UNITED STATES

## Abstract

**Background:**

Hypoxic microenvironments are generated during fungal infection. It has been described that to survive in the human host, fungi must also tolerate and overcome *in vivo* microenvironmental stress conditions including low oxygen tension; however nothing is known how *Paracoccidioides* species respond to hypoxia. The genus *Paracoccidioides* comprises human thermal dimorphic fungi and are causative agents of paracoccidioidomycosis (PCM), an important mycosis in Latin America.

**Methodology/Principal Findings:**

In this work, a detailed hypoxia characterization was performed in *Paracoccidioides*. Using NanoUPLC-MS^E^ proteomic approach, we obtained a total of 288 proteins differentially regulated in 12 and 24 h of hypoxia, providing a global view of metabolic changes during this stress. In addition, a functional characterization of the homologue to the most important molecule involved in hypoxia responses in other fungi, the SREBP (sterol regulatory element binding protein) was performed. We observed that *Paracoccidioides* species have a functional homologue of SREBP, named here as SrbA, detected by using a heterologous genetic approach in the *srbA* null mutant in *Aspergillus fumigatus*. *Paracoccidioides srbA* (*PbsrbA*), in addition to involvement in hypoxia, is probable involved in iron adaptation and azole drug resistance responses.

**Conclusions/Significance:**

In this study, the hypoxia was characterized in *Paracoccidioides*. The first results can be important for a better understanding of the fungal adaptation to the host and improve the arsenal of molecules for the development of alternative treatment options in future, since molecules related to fungal adaptation to low oxygen levels are important to virulence and pathogenesis in human pathogenic fungi.

## Author Summary

The genus *Paracoccidioides* is composed of species that are causative agents of paracoccidioidomycosis (PCM), a neglected human granulomatous mycosis, endemic in Latin America. To survive in the human host, fungi must tolerate and overcome *in vivo* micro environmental stress conditions, including low oxygen levels. *Paracoccidioides* spp. depicts differential responses to several stresses such as iron/zinc deprivation, oxidative and nitrosative stresses and carbon starvation. In addition, *Paracoccidioides* yeast cells recovered from liver of infected mice demonstrated adaptability to the host conditions. Mechanisms by which fungi sense oxygen levels have been characterized, although this is the first description in *Paracoccidioides* spp. Little is known about hypoxia in thermally dimorphic fungi and nothing has been studied in *Paracoccidioides* genus, one of the representatives of this group of pathogens. A detailed characterization of the hypoxia responses was performed using proteomic and heterologous genetics approaches. *Paracoccidioides* genus have a functional homologue of the key regulator of hypoxia adaptation in fungi, SrbA, a SREBP (sterol regulatory element binding protein) orthologue. The proteome during hypoxia provided a global view of metabolic changes during this stress and species of the *Paracoccidioides* genus have a functional SrbA. Our study provides a better understanding of the fungal adaptation to the host and it can improve the arsenal of molecules for the development of alternative treatment options to paracoccidioidomycosis, since molecules related to fungal adaptation to low oxygen levels are important to virulence and pathogenesis in human pathogenic fungi.

## Introduction

The genus *Paracoccidioides* is a complex of thermodimorphic fungi, and are causative agents of paracoccidioidomycosis (PCM) a deep systemic granulomatous mycosis, endemic in Latin America [1, 2]. *Paracoccidioides* spp. grows as yeast in host tissue and *in vitro* at 36°C, and as mycelium under saprobiotic and laboratory conditions (18–23°C). As the dimorphism is dependent on temperature, when the mycelia or conidia are inhaled into the host respiratory tract, the transition to the pathogenic yeast phase occurs [3]. Once in the lungs, epithelial cells and resident macrophages are the first line of defence against *Paracoccidioides* cells. Inside macrophages, the parasitic yeast form subverts the normally harsh intraphagosomal environment and proliferates [4]. Adhesion to and invasion of epithelial cells and basal lamina proteins may be required for the extra pulmonary haematogenous fungal dissemination to organs and tissues [1, 3, 5].

To survive in the human host, fungi must also tolerate and overcome *in vivo* micro environmental stress conditions. Conditions such as high temperature, distinct ambient pHs, carbon and metal ions deprivation, and gas tension (high levels of carbon dioxide and low oxygen levels) induce several stress responses in the invading fungus [6–10]. In *Paracoccidioides* spp., previous analyses have demonstrated differential responses to iron and zinc deprivation, oxidative and nitrosative stress and carbon starvation faced by the fungus during infection [11–15]. In addition, *Paracoccidioides* spp. yeast cells recovered from liver of infected mice and from infected macrophages alter their metabolism in order to adapt to the host using available nutrition sources [16, 17].

It is well established that oxygen levels vary throughout the mammalian body depending on numerous factors including tissue type and presence or absence of an inflammatory response [18]. Oxygen levels in most mammalian tissues are found to be considerably below atmospheric levels (21%) [19, 20]. Also, oxygen availability at the sites of inflammation is significantly reduced compared to surrounding tissues [21, 22] since, in inflamed tissues, the blood supply is often interrupted because the vessels are congested with phagocytes or the pathogen itself [23, 24]. Thus, it seems highly probable that hypoxic microenvironments are generated during fungal infection [25, 26].

Mechanisms used by fungi to sense oxygen levels have been characterized [27]. An SREBP (sterol regulatory element binding protein) ortholog, previously characterized in higher eukaryotes [28–32], was first identified and characterized in the fission yeast *Schizosaccharomyces pombe* as an oxygen sensor [33, 34]. Later, it was characterized in the human pathogenic fungi *Cryptococcus neoformans* and *Aspergillus fumigatus* [35–37]. In *A*. *fumigatus*, the SREBP homologue, SrbA, controls the expression of genes involved in biosynthesis of lipids, ergosterol, and heme [37, 38]. Recently, a new transcriptional regulator of the fungal hypoxia response and virulence that genetically interacts with SrbA, named SrbB, was also characterized in *A*. *fumigatus* [39]. In *S*. *pombe* and *C*. *neoformans* the SREBP homologues also regulate enzymes in the ergosterol biosynthetic pathway under hypoxic conditions [34, 35, 38].

Oxygen levels are low in subsurface layers of organic matter in natural environments that are habitats of environmental pathogens such *Paracoccidioides* and *Aspergillus* [40–43]. In this context, studies regarding the responses of *Paracoccidioides* to hypoxia are of relevance and in this study are described for the first time. Up to now, hypoxia has not been described in the *Paracoccidioides* genus, representatives of thermally dimorphic fungi, in which responses to hypoxia remain to be investigated. We observed that *Paracoccidioides* yeast cells respond to hypoxia regulating the expression of proteins from diverse metabolic pathways. We also observe that species of the *Paracoccidioides* genus have homologues of the key regulator of hypoxia adaptation in fungi, SrbA. *Paracoccidioides srbA* was characterized using a heterologous genetics approach that confirmed the functional conservation of this protein in the hypoxia response. *Paracoccidioides srbA* (*PbsrbA*) is likely involved in hypoxia, iron adaptation and azole drug resistance responses, as observed by functional complementation of the *srbA* null mutant in *A*. *fumigatus* by *PbsrbA*. The obtained data, may improve the arsenal of molecules for the development of alternative treatment options since molecules related to fungal adaptation to low oxygen levels are important to virulence and pathogenesis in human pathogenic fungi.

## Methods

### *Paracoccidioides* and *A*. *fumigatus* maintenance and hypoxia cultivation

*Paracoccidioides*, *Pb*01 (ATCC MYA-826), was used in the experiments. The yeast phase was cultivated for 7 days, at 36°C in BHI semisolid medium added of 4% (w/v) glucose. When required, the cells were grown for 72 h at 36°C in liquid BHI, washed with PBS 1X, and incubated at 36°C in McVeigh/Morton (MMcM) medium as previously described [44]. *Pb*01 yeast cells were subjected to normoxia and hypoxia as previously described [37, 45]. Normoxia was considered general atmospheric levels within the lab (~21% O_2_). For hypoxia, an incubator (Multi-Gas Incubator MCO-19M-UV, Panasonic Biomedical) was used. The chamber was maintained at 36°C, and kept at 1% oxygen level, utilizing a gas mixture containing 1% O_2_, 5% CO_2_ and 94% N_2_. *Paracoccidioides* yeast cells viability was determined as previously described: the number of viable cells was determined at times of 0, 6, 12, 18 and 24 h by staining with 0.01% (w/v) trypan blue in PBS1X [12, 46, 47].

All *A*. *fumigatus* strains were routinely grown in glucose minimal medium (GMM) with appropriate supplements at 37°C as previously described [45, 48]. To prepare solid media, 1.5% (w/v) agar was added, before autoclaving. For protein extraction and associated mRNA abundance experiments, 0.5% (w/v) yeast extract was added to liquid GMM to increase hypha mass [45]. For hypoxia cultivations, an incubation chamber (Invivo_2_ 400; Ruskinn) was used. The chamber was maintained at 37°C and kept at 1% O_2_, 5% CO_2_, and 94% N_2_, controlled through a gas mixer (Gas Mixer Q; Ruskinn/Baker Company). Normoxia was also considered general atmospheric levels within the lab (~21% O2).

### Sample preparation, NanoUPLC-MS^E^ data acquisition and processing and protein identification

Following *Paracoccidioides* yeast cells incubation under normoxia and hypoxia, in biological triplicates, cells were centrifuged at 1,500 x *g*, resuspended in 50 mM ammonium bicarbonate pH 8.5 and disrupted using glass beads and bead beater apparatus (BioSpec, Oklahoma, USA) in 5 cycles of 30 sec, while on ice. The cell lysate was centrifuged at 10,000 x *g* for 15 min at 4°C and the supernatants for each condition were polled in equimolar amounts and subjected to the nanoscale liquid chromatography coupled with tandem mass spectrometry in 3 technical replicates. The proteins were quantified using the Bradford reagent (Sigma-Aldrich) [49]. Sample aliquots (70 μg) were prepared for NanoUPLC-MS^E^ as previously described [11, 15, 50, 51], with some modifications. Briefly, 50 mM ammonium bicarbonate was added and was followed by addition of 35 μL of RapiGEST (0.2%v/v) (Waters Corp, Milford, MA). The solution was vortexed and then incubated at 80°C for 15 min. The disulphide bonds were reduced by treating proteins with 10mM D-L-dithiothreitol for 30 min at 60°C. The sample was cooled at room temperature and the proteins were alkylated with 200 mM iodoacetamide in a dark room for 30 min. Proteins were digested with trypsin (Promega, Madison,WI, USA, 1:25 w/v) prepared in 50 mM ammonium bicarbonate, at 37°C overnight. Following the digestion, 10 μL of 5% (v/v) trifluoroacetic acid was added to hydrolyse the RapiGEST, followed by incubation at 37°C for 90 min. The sample was centrifuged at 18,000 x *g* at 6°C for 30 min, and the supernatant was transferred to a Waters Total Recovery vial (Waters Corp). A solution of one pmol.ul^-1^ MassPREP Digestion Standard [rabbit phosphorylase B (PHB)] (Waters Corp) was used to prepare the final concentration of 150 fmol.ul^-1^ of the PHB. The buffer solution of 20 mM ammonium formate (AF) was used to increase the pH. The digested peptides were separated further via NanoUPLC-MS^E^ and analysed using a nanoACQUITY system (Waters Corporation, Manchester, UK). Mass spectrometry data obtained from NanoUPLC-MS^E^ were processed and searched against the *Paracoccidioides* database (http://www.broadinstitute.org/annotation/genome/paracoccidioides_brasiliensis/MultiHome.html) using ProteinLynx Global Server (PLGS) version 2.4 (Waters Corp). Protein identifications and quantitative data packaging were performed using dedicated algorithms [52, 53]. The ion detection, clustering, and log-scale parametric normalizations were performed in PLGS with an ExpressionE license installed (Waters, Manchester, UK). The false positive rate (FPR) of the algorithm for protein identification was set to 4% in at least two out of three technical replicate injections. Using protein identification replication as a filter, the false positive rate was minimized because false positive protein identifications, i.e., chemical noise, have a random nature and do not tend to replicate across injections. For the analysis of the protein identification and quantification level, the observed intensity measurements were normalized to the intensity measurement of the identified peptides of the digested internal standard. Normalization was performed with a protein that showed no significant difference in abundance in all injections [54] to accurately compare the expression protein level to normoxia and hypoxia samples. For 12 and 24 h, the proteins oxidoreductase 2-nitropropane dioxygenase and 40S ribosomal protein S5 were used as normalizing proteins, respectively (PAAG_01321 and PAAG_05484 from *Paracoccidioides* genome database http://www.broadinstitute.org/annotation/genome/paracoccidioides_brasiliensis/MultiHome.html). Furthermore, only those proteins with a fold change higher than 50% difference were considered to be expressed at significantly induced/ repressed levels.

### Mitochondrial activity in *Paracoccidioides* yeast cells under hypoxia

*Paracoccidioides*, *Pb*01 yeast cells, were grown under normoxia and hypoxia for 12 and 24 h, in biological triplicates. Following that, cells were harvested by centrifugation at 2,000 x *g* for 5 min at 4°C and diluted in PBS buffer at 10^6^ cells/ml. Cells were stained with Rhodamine 123 (1.2 mM) (Sigma Aldrich) according to the manufacturer's protocol and then washed twice with 1X PBS. Stained cells were observed under a fluorescence microscope (AxioScope A1, Carl Zeiss) and analysed with the 546–512 nm filter. Rhodamine fluorescence intensity was measured using the AxioVision Software (Carl Zeiss). The minimum of 100 cells for each microscope slides, in triplicates, for cells submitted to hypoxia and normoxia for 12 and 24 h were used to measure the rhodamine fluorescence intensity. The software provided the fluorescence intensity (in pixels) and the standard deviation of each analysis. Statistical comparisons were performed using the student’s t test and p-values ≤ 0.05 were considered statistically significant.

### *In silico* analysis of SREBPs orthologs

The amino acid predicted sequences were obtained from GenBank (http://www.ncbi.nlm.nih.gov/) to *Paracoccidioides Pb*01 (XP_002794199); *Pb*03 (KGY15961); *Pb*18 (EEH47197); *Aspergillus fumigatus* (XP_749262); *Schizosaccharomyces pombe* (NP_595694); *Cryptococcus neoformans* (XP_567526) and *Homo sapiens* (P36956). The SMART tool (http://smart.embl-heidelberg.de) [55, 56] was used to search for conserved domain bHLH (*basic helix-loop- helix leucine zipper DNA-binding domain*) and Phobius (http://phobius.sbc.su.se/) [57] and SACS MEMSAT2 Prediction software (http://www.sacs.ucsf.edu/cgi-bin/memsat.py) [58] were used to depict transmembrane segments. The amino acid sequences from all proteins were aligned using CLUSTALX2 [59] to show a conserved tyrosine residue (indicated by asterisk) specific to the SREBP family of bHLH transcription factors.

### RNA extraction and quantitative real time PCR (qRT-PCR)

Following *Paracoccidioides* incubation under hypoxia and normoxia, cells were harvested, and total RNA was extracted using TRIzol (TRI Reagent, Sigma-Aldrich, St. Louis, MO, USA) and mechanical cell rupture (Mini-Beadbeater-Biospec Products Inc., Bartlesville, OK). Total RNA was extracted and treated with DNase (RQ1 RNase-free DNase, Promega). After in vitro reverse transcription (SuperScript III First-Strand Synthesis SuperMix; Invitrogen, Life Technologies), the cDNAs were submitted to a qRT-PCR reaction, which was performed using SYBR Green PCR Master Mix (Applied Biosystems, Foster City, CA) in a StepOnePlus Real-Time PCR System (Applied Biosystems Inc.). The expression values were calculated using the transcript that encoded alpha tubulin (GenBank accession number XP_002796639) as the endogenous control, as previously reported [11] and, when required, the data were presented as relative expression in comparison to the experimental control cells value set at 1. Relative expression levels of genes of interest were calculated using the standard curve method for relative quantification [60]. Briefly, for each of the three replicates of a sample, the average quantity (avg) was calculated of target cDNA interpolated from the standard curve, the standard deviation of the average (stdev), and the coefficient of variation (CV) according to the formula CV = stdev/ avg. Any outlier points (>17% CV) was removed and avg, stdev and CV were recalculated. For each sample, the gene of interest (GOI) was normalized to the reference gene (RG) for the sample according to the following equation: normalized value = avg GOI quantity/ avg RG quantity. The standard deviation (SD) of the normalized value was calculated according to the equation: SD = (normalized value) x square root (CV reference gene + CV gene of interest)^2^. The resulting values were plotted as a bar graph of normalized value versus sample name or experimental treatment group, with the error bars equal to the SD, of the biological triplicates of independent experiments [60]. Standard curves were generated by diluting the cDNA solution 1:5. Statistical comparisons were performed using the student’s t test and p-values ≤ 0.01 were considered statistically significant.

Regarding to *A*. *fumigatus*, wild type, *ΔsrbA* and reconstituted strains were cultured in liquid GMM under normoxia or hypoxia. Germlings and mycelia were collected with vacuum filtration and lyophilized, prior to homogenization with 0.1-mm glass beads. Total RNA was extracted, treated with DNase, reversed transcripted to cDNA and submitted to a qRT-PCR reaction, identically to which was performed to *Paracoccidioides*. Oligonucleotides to amplify the *srbA* gene from *A*. *fumigatus* and *Paracoccidioides* were used in the experiments. The data were normalized using the *A*. *fumigatus tefA* reference gene [61]. Primers are depicted in S3 Table.

### Genetic complementation assay in *A*. *fumigatus*

The *A*. *fumigatus* strains CEA10 (wild type) and a *srbA* null mutant of *A*. *fumigatus* were used in the genetic complementation assays. This *srbA* null mutant was previously generated by replacement of the *srbA* coding sequence in *A*. *fumigatus* strain CEA17 with the auxotrophic marker *pyrG* from *A*. *parasiticus* as previously described [45, 62, 63]. To perform genetic complementation of the respective *ΔsrbA*, the *Paracoccidioides Pb*01 *srbA* sequence was amplified from *Paracoccidioides* genomic DNA as template and linked together with a fragment of the *gpdA* (glyceraldehyde phosphate dehydrogenase) gene from *Aspergillus nidulans*, used as promoter and a functional *pyrG* gene from *A*. *parasiticus*, used to select the transformed strains (*gpdA*+*PbsrbA+pyrG*). The fused product was used to perform fungal transformations. Generation of fungal protoplasts and polyethylene glycol-mediated transformation of *A*. *fumigatus* were performed as previously described [45, 64]. Reconstituted strains were confirmed by screening using hypoxia chamber, conventional PCRs, Southern blots, qRT-PCRs and immunoblot analyses. All primers used are shown in S3 Table. In order to eliminate the chance of heterokaryons, each transformant was streaked with sterile toothpicks a minimum of twice, to obtain colonies from single conidia. All strains were stored as frozen stocks with 50% (v/v) glycerol at -80°C.

### Immunoblots

Ten-well 10% Mini-Protean precast gel (Bio-Rad) was used for SDS-PAGE. Denatured protein was loaded (40 μg per well). After gel electrophoresis, protein was transferred to a nitrocellulose membrane (Hybond-C Extra; Amersham Biosciences). *Pb*SrbA was detected on blots using the *A*. *fumigatus* SrbA 1–275 recombinant primary N-terminus antibody at a 1/27,000 dilution and an anti-rabbit alkaline phosphatase (AP)-conjugated secondary antibody raised in goat (Abcam) at a 1/5,000 dilution, as previously described [45]. Chemiluminescence was measured following incubation of blots with Tropix CPD Star substrate (Applied Biosystems) with Immun-star enhancer (Bio-Rad) using a FluorChem FC2 imager (Alpha Innotech).

### Southern blots

DNA was isolated from overnight liquid cultures of *A*. *fumigatus*. The mycelium was separated from the medium by filtration and glass beads were used to disruption. Additional purification steps were used to isolate the genomic DNA and Southern blot was performed using the digoxigenin labelling system (Roche Molecular Biochemicals, Mannheim, Germany) as previously described [45, 65]. Briefly, 30 μg aliquots of genomic DNA were digested with *Hind*III and *Eco*RI to detect *gpdA* and *pyrG*, respectively. Restriction digests were separated on a 1% agarose gel and blotted onto nylon membranes. The concentration of the probes in hybridization solution was 50 ng/ml, and hybridization was carried out at 50°C. Membranes were washed in a final solution of 0.1 SSC and 0.1% (w/v) sodium dodecyl sulphate, at 68°C.

### Iron depletion experiments

Production of biomass was performed to wild type, *ΔsrbA* and reconstituted strain 1 (Rec 1) of *A*. *fumigatus*. A total of 10^8^ cells of each strain were grown under iron starvation (−Fe) and iron sufficiency (0.03 mM, +Fe) in liquid medium for 24 h, at 37°C. The cells were harvested by vacuum filtration and then lyophilized. The data represent the mean ± SD of biological triplicates and the values were normalized to the reconstituted strain. Statistical comparisons were performed using the student’s t test and p-values ≤ 0.01 were considered statistically significant.

*P*. *brasiliensis* yeast cells were grown in McVeigh/Morton medium (MMcM) [44] and the yeast cells were incubated at 36°C with shaking at 150 rpm. In order to analyse the kinetic of expression of *PbsrbA*, the cells were cultivated under iron deprivation or supplementation, using the iron chelator bathophenanthroline disulfonate (BPS; 50 μM; Sigma-Aldrich, Germany) or 3.5 μM Fe(NH4)2(SO4)2, respectively. Total RNA was extracted at 30 min, 1, 3 and 24 h and the quantitative real time PCR was performed as cited above.

## Results and Discussion

### Overview of hypoxia regulated proteins in *Paracoccidioides Pb*01

In order to start the characterization of *Paracoccidioides* hypoxia response, we utilized a proteomics approach. The NanoUPLC-MS^E^ [50, 51] was previously used to map metabolic changes in *Paracoccidioides* at a protein level [11, 13, 15, 17] and was also used in this study. After exposing the cells to normoxia (21% pO_2_) and hypoxia (1% pO_2_) and using a proteomics approach at time points 12 and 24 h, we observed significant differences in protein expression indicating that the fungus responds to hypoxia.

**Fig 1:**
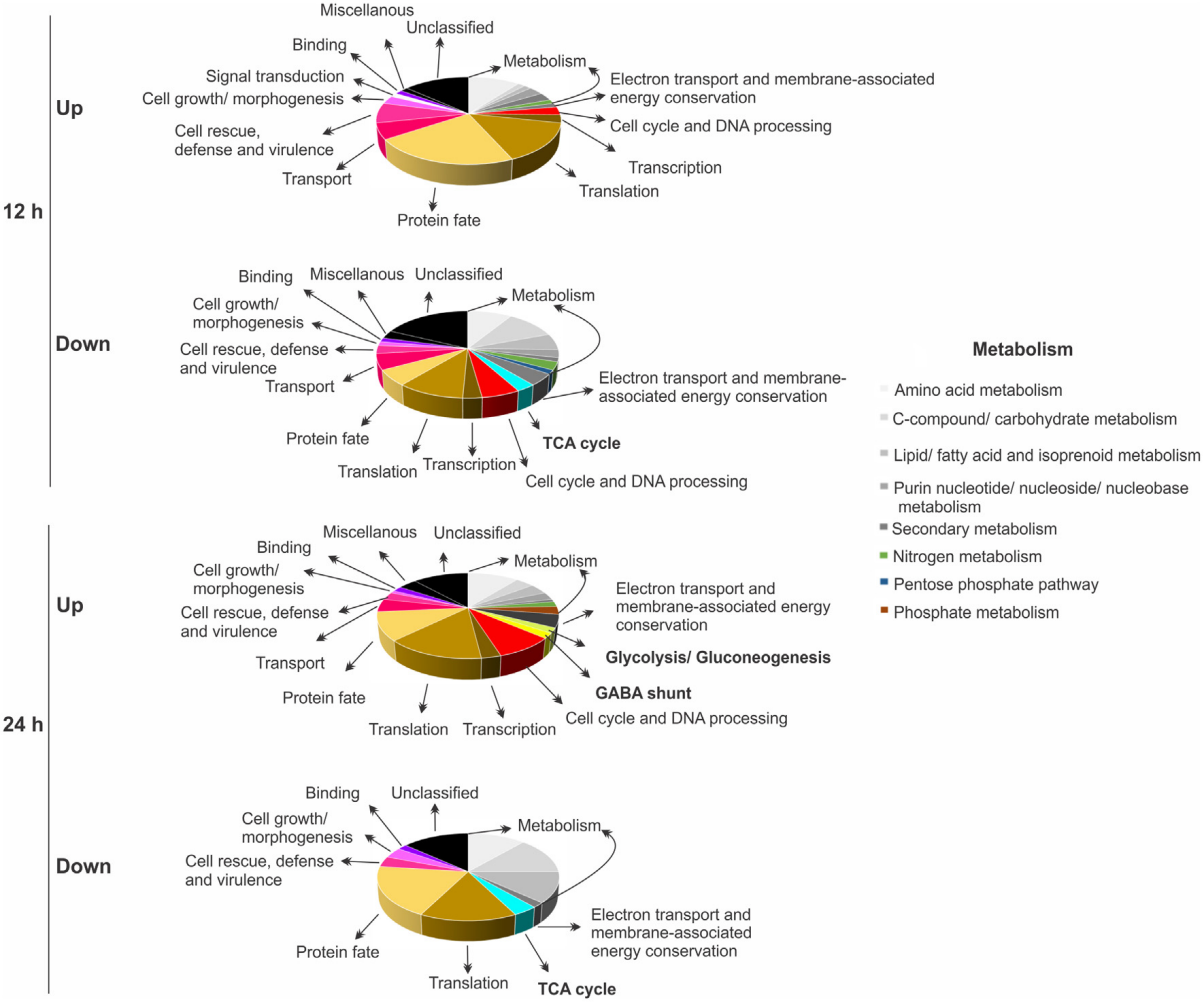
Functional classification of proteins regulated in *Paracoccidioides* upon hypoxia obtained by NanoUPLC-MS^E^ data. Biological processes of differentially expressed proteins (up- and down-regulated) in *Paracoccidioides*, *Pb*01, submitted to 12 and 24 h of hypoxia are shown. The biological processes were obtained using the Pedant on MIPS (http://pedant.helmholtz-muenchen.de/pedant3htmlview/pedant3view?Method=analysis&Db=p3_r48325_Par_brasi_Pb01) and Uniprot databases (http://www.uniprot.org/). One hundred and thirty four and 154 proteins were differentially expressed in 12 and 24 h under hypoxia, respectively, compared with normoxia. In 12 h, 67 proteins were induced and the same number repressed while in 24 h, 102 proteins were induced and 52 were repressed under the same conditions.

As described in Lima and co-workers [15], a 1.5-fold change was used as a threshold to determine positively and negatively differentially proteins. In total, 134 and 154 proteins presented different abundances in 12 and 24 h under hypoxia, respectively, compared to normoxia. In 12 h, the same number of proteins (67) were increased and decreased, upon hypoxia, compared to control (normoxia). At 24 h, 102 proteins were increased and 52 were decreased (S1 and S2 Tables). The adaptation mechanism of *Paracoccidioides* to hypoxia, as represented by biological processes, as deduced from increased and decreased proteins is shown in Fig 1. Proteins associated with several subcategories of metabolism were represented in both analyses as increased and decreased proteins. Some of them were less represented in 24 h of hypoxia such as nitrogen, purine nucleotide/ nucleoside/ nucleobase and phosphate metabolism. Proteins associated with energy depicted an interesting profile of abundance. Those involved with electron transport/ membrane associated energy conservation were enriched for reduced levels in 12 h of hypoxia, and levels were subsequently restored at 24 h of hypoxia (Fig 1, S1 and S2 Tables).

**Fig 2:**
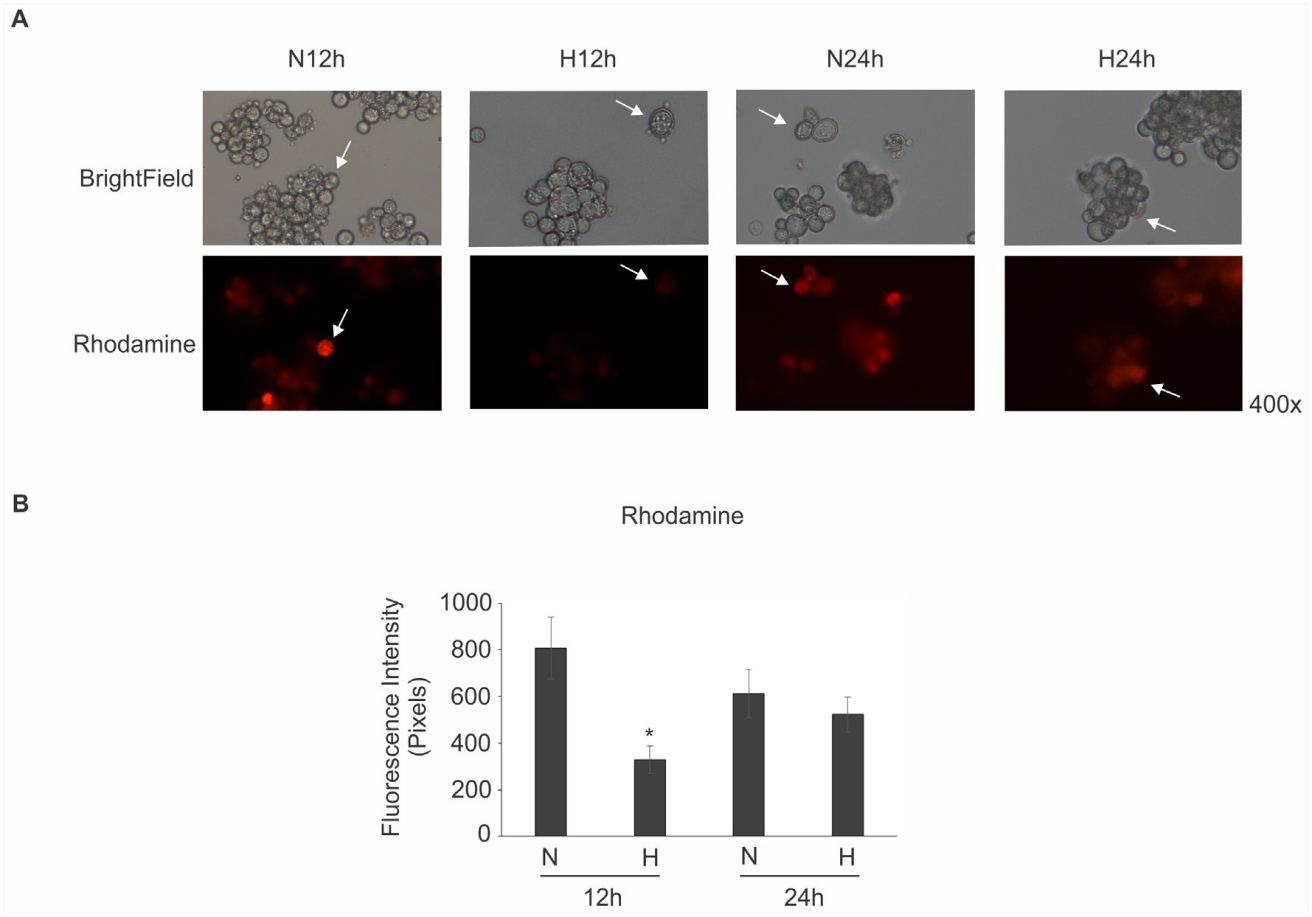
Mitochondrial activity of *Paracoccidioides* submitted to normoxic and hypoxic stress. **(A)** *Pb*01 yeast cells were grown in BHI medium in normoxia (N), 21% pO_2_, and hypoxia (H), 1% pO_2_, for 12 and 24 h. The mitochondrial activity was evaluated by using rhodamine as a dye for mitochondrial membrane potential. The lower intensity of mitochondria activity was detected in 12 h of hypoxia. The white arrows indicates representative cells from population. **(B)** The rhodamine fluorescence intensity of cells grown in normoxia (N) and hypoxia (H) for 12 and 24 h was measured using the AxioVision Software (Carl Zeiss). The values of fluorescence intensity (in pixels) and the standard deviation of each analysis were used to plot the graph. Data are expressed as mean ± standard deviation (represented using error bars) of the minimum of 100 cells for each microscope slide, in triplicates, for each condition. *, significantly different comparison with normoxia condition, at a P value of ≤ 0.05.

To further assess this observation, we evaluated mitochondrial activity using rhodamine, a permeable lipophilic cationic fluorescent probe that accumulates in mitochondria and is distributed electrophoretically into the mitochondrial matrix in response to mitochondrial electric potential [66, 67]. The rhodamine probe has been used to stain yeast cells [67], including *Paracoccidioides* [13, 68]. Consistent with the proteomics data that suggested reduced mitochondrial activity, a low level of staining of rhodamine was observed in yeast cells during 12 h of hypoxia. At 24 h, the intensity of detection was restored, which is consistent with proteomics data (Fig 2). Additionally, proteins such as catalase, thioredoxin, chaperones and gamma-glutamyltranspeptidase were up-regulated in *Paracoccidioides* in hypoxia for 12 h (S1 Table) and could be associated with the altered mitochondrial activity. Our suggestion is that the fungus possibly induces ROS scavenging enzymes to protect the fungus against low oxygen effects that induces a strong reduction in electron-transfer reactions. In *C*. *neoformans*, several genes associated with the mitochondrial activity were identified as essential for hypoxic growth [69].

Proteins in the energy subcategories glycolysis/ gluconeogenesis, TCA cycle and GABA shunt were also differentially abundant under hypoxia. The glycolysis/ gluconeogenesis and GABA shunt were increased at 24 h of hypoxia. On the other hand, proteins of the TCA cycle were reduced at both time points (Fig 1, S1 and S2 Tables). The mechanisms of hypoxia adaptation are variable among fungi [18, 70]. At transcript level, for example, genes involved with glycolysis were induced, while those involved with aerobic respiration were repressed in *Candida albicans*, a facultative anaerobe, submitted to hypoxia [71–73]. However, in the obligate aerobic yeast *C*. *neoformans*, a general lack of changes in glycolytic mRNA abundance was observed in response to hypoxia, and genes involved in mitochondrial function have been observed to be critical for the hypoxia response [36, 74]. In the obligate aerobic mold *A*. *nidulans*, exposure to hypoxia results in an increase in glycolytic gene transcripts and the GABA shunt, which bypasses two steps of the tricarboxylic acid (TCA) cycle [75]. Transcriptome data from *A*. *nidulans* largely correlated with the proteomic profile, in which proteins in core metabolism and utilization of the GABA shunt was identified [76]. Similar results were found in *A*. *fumigatus*, upon short-term hypoxia as the GABA shunt was also induced [77]. On the other hand, cultures exposed to long-term hypoxia revealed increased abundance of proteins involved in glycolysis, respiration, pentose phosphate pathway, and amino acid and pyruvate metabolism [78].

**Fig 3:**
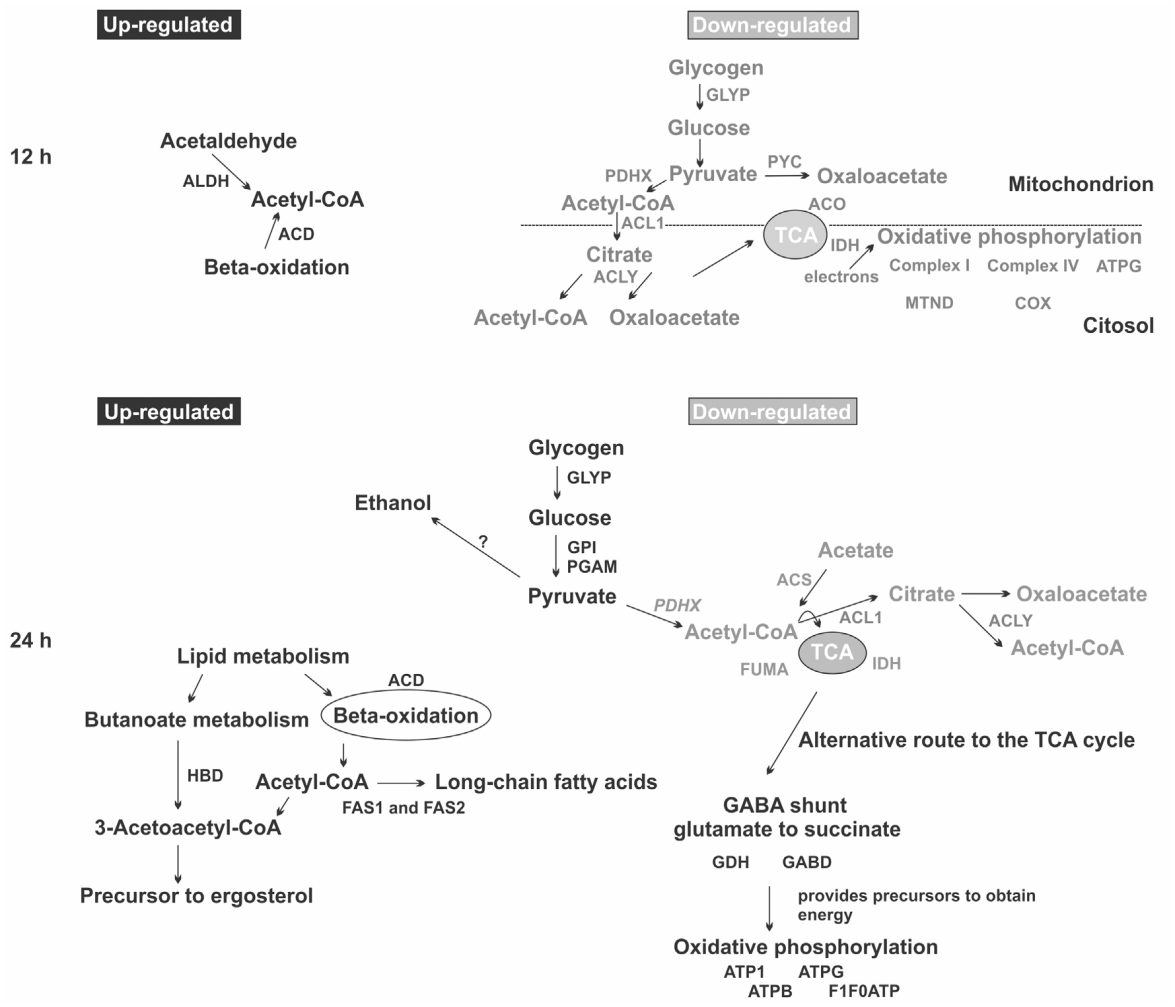
Overview of metabolic responses of the *Paracoccidioides* upon hypoxia. The figure summarizes the data from proteomic analysis and shows the main changes in *Paracoccidioides Pb*01 metabolism during hypoxia, for 12 and 24 h. Up-regulated proteins detected at both time points are indicated by black, and down-regulated proteins, by grey colours. Proteins are indicated by letters: **ACD** [long-chain specific acyl-CoA dehydrogenase]; **ALDH** [aldehyde dehydrogenase]; **GLYP** [glycogen phosphorylase]; **PYC** [pyruvate carboxylase]; **PDHX** [pyruvate dehydrogenase protein X component]; **ACL1** [ATP-citrate synthase subunit 1]; **ACLY** [ATP-citrate lyase]; **ACO** [aconitate hydratase]; **IDH** [isocitrate dehydrogenase]; **MTND** [NADH-ubiquinone oxidoreductase]; **COX** [cytochrome-c oxidase chain VI]; **ATPG** [ATP synthase gamma chain]; **GPY** [glucose-6-phosphate isomerase]; **PGAM** [2,3-bisphosphoglycerate independent phosphoglycerate mutase]; **HBD** [3-hydroxybutyryl-CoA dehydrogenase]; **FAS1** [fatty acid synthase subunit beta dehydratase]; **FAS2** [fatty acid synthase subunit alpha reductase]; **GDH** [NADP specific glutamate dehydrogenase]; **GABD** [succinate-semialdehyde dehydrogenase]; **ATP1** [ATPase alpha subunit]; **ATPB** [ATP synthase subunit beta]; **F1F0ATP** [mitochondrial F1F0 ATP synthase subunit]; **ACS** [acetyl-coenzyme A synthetase]; **FUMA** [fumarate hydratase].

Fig 3 depicts probable mechanisms used by *Paracoccidioides* to overcome hypoxic environments. It does not represent an integral model of how *Paracoccidioides* adapts to hypoxia, but from our point of view is an important source to start the understanding of how this fungus adapts to low oxygen levels. The abundance of some enzymes involved in acetyl-CoA production are up-regulated in 12 h of hypoxia compared to normoxia. The induction, for example, of the aldehyde dehydrogenase and long-chain specific acyl-CoA dehydrogenase enzymes suggest that the acetyl-CoA is produced via acetaldehyde and beta-oxidation pathway, respectively. Consistent with these data, proteins involved in glycolysis were decreased in abundance (S2 Table). Acetyl-CoA can be used as an alternative carbon source under these conditions (Fig 3). In fact, the expression of proteins related to glycolysis, acetyl-CoA production from pyruvate and citrate, TCA cycle and oxidative phosphorylation were reduced (Fig 3, S2 Table).

At 24 h, the detected up- and down-regulated proteins could show additional changes in *Paracoccidioides* strategies to adapt to hypoxia. For example, proteins involved in glycolysis are now increased supporting pyruvate production. In addition, the GABA shunt is increased at 24 h of hypoxia (Fig 3, S1 Table). The increased abundance of two enzymes involved with the GABA shunt pathway, NADP specific glutamate dehydrogenase and succinate-semialdehyde dehydrogenase, support this hypothesis in *Paracoccidioides*. Reports have shown that GABA is generated from 2-oxoglutarate *via* glutamate through the actions of glutamate dehydrogenase and glutamate decarboxylase, and that GABA transaminase irreversibly transaminates GABA to succinic semialdehyde, which is then oxidized to succinate by succinic semialdehyde dehydrogenase [76, 79, 80]. Transcripts for this pathway are also up-regulated in *A*. *nidulans* and *A*. *fumigatus*, under hypoxia [75, 77]. The GABA shunt is hypothesized to help organisms to avoid accumulation of high NADH levels in the absence of a terminal electron acceptor such as oxygen, and also contributes to glutamate formation [77]. This pathway is also described as an alternative route to the TCA cycle [75]. Interesting, the TCA pathway was down-regulated, based on protein levels of key enzymes (Fig 3, S2 Table), although the role of the GABA shunt in the fungal hypoxia response remains to be conclusively determined.

**Fig 4:**
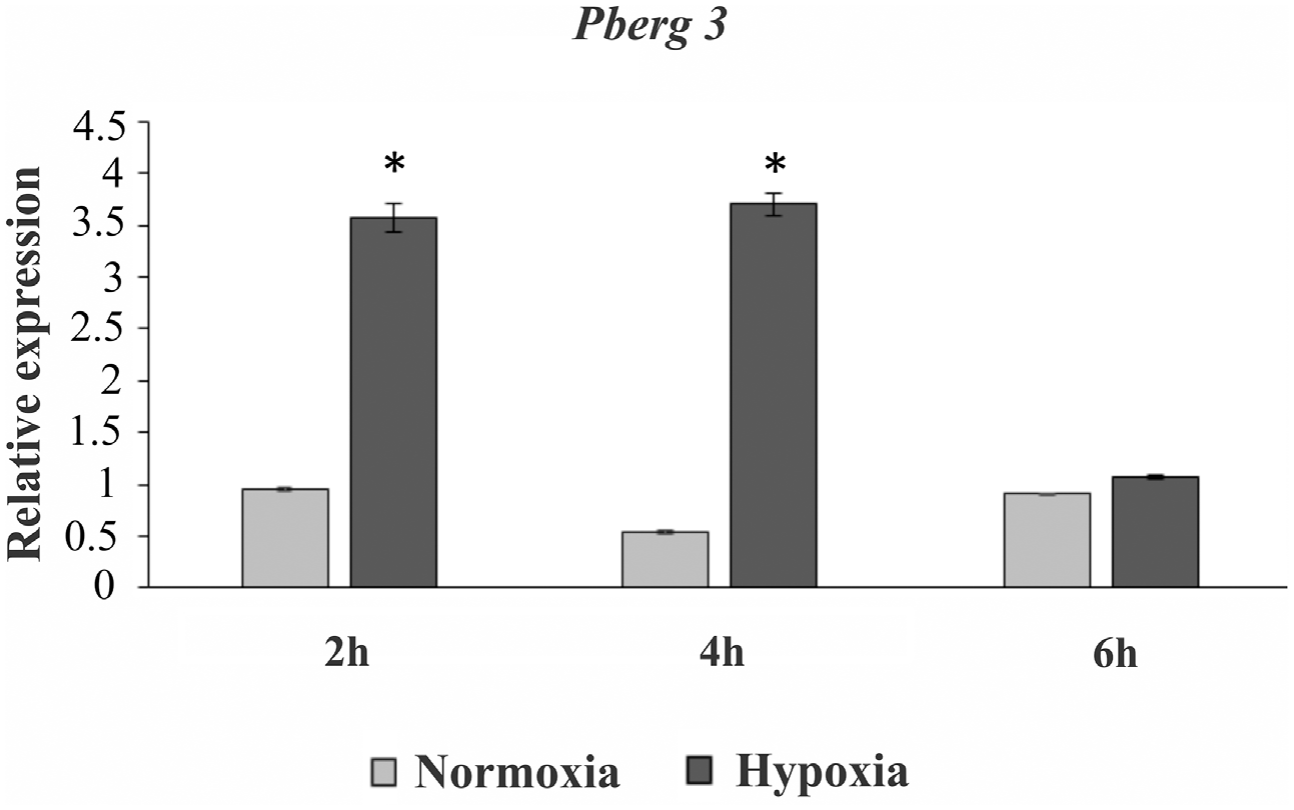
Kinetic of expression of a representative gene of ergosterol pathway in *Paracoccidioides* submitted to hypoxia. The kinetic of expression of *Pberg3*, representative gene of ergosterol pathway, was analysed using qRT-PCR. *Pb*01 yeast cells were submitted to normoxia and hypoxia for 2, 4 and 6 h at 37°C in BHI medium and total RNAs were extracted. Molecules of cDNA were synthesized and used for qRT-PCR. The data were normalized using the constitutive gene encoding the alpha tubulin as the endogenous control. Data are expressed as the mean ± standard deviation of the triplicates of independent experiments. *, significantly different from the normoxic condition (experimental control), at a p value of P ≤ 0.01. The accession number to *erg3* is PAAG_03651, from *Paracoccidioides* genome database (http://www.broadinstitute.org/annotation/genome/paracoccidioides_brasiliensis/MultiHome.html).

Moreover, enzymes involved in beta-oxidation and in production of ergosterol precursor molecules were also up-regulated according to proteomic data, at 24 h (Fig 3, S1 Table). During *Paracoccidioides* hypoxia adaptation, the detection of the long-chain specific acyl-CoA dehydrogenase, for example, shows that the fungus activates the beta-oxidation resulting in acetyl-CoA, that could be involved in fatty acid and ergosterol production. The enzyme 3-hydroxybutyryl-CoA dehydrogenase yields 3-acetoacetyl-CoA that together to acetyl-CoA supports ergosterol synthesis. Our suggestion makes sense since acetyl-CoA is probably not produced by pyruvate, neither acetate nor citrate, since enzymes related to their metabolism are down regulated in our data (Fig 3, S2 Table). The relative expression level of the transcript encoding *Pberg3* was determined by quantitative real time PCR (Fig 4). The gene *Pberg3* encodes C-5 sterol desaturase, an enzyme involved in the late steps in sterol biosynthesis [74, 81]. The data provide additional evidence that *Paracoccidioides* faces hypoxia and regulates ergosterol production, to compensate the effects of low oxygen levels. Several enzymatic steps in ergosterol biosynthesis are catalysed by iron and oxygen-requiring enzymes including that performed by Erg3 [74]. Also, the metabolism of fatty acids and ergosterol are increased in *C*. *albicans*, *C*. *neoformans*, *A*. *fumigatus* and *A*. *nidulans* in response to hypoxia and these molecules are required for the stability, fluidity and structure of the fungus plasma membrane [36, 72–74, 76, 77]. On this way, the fungus might be remodelling the fatty acid content of membrane lipids to keep the membrane fluidity in hypoxia. Along with ergosterol’s role as a target to antifungal drugs, the understanding of the mechanisms that regulate ergosterol biosynthesis is of interest to biomedical research [82, 83]. In *S*. *pombe*, *A*. *fumigatus* and *C*. *neoformans*, the SREBP proteins are effectors which sense changes in oxygen levels indirectly through alterations in ergosterol levels [33, 35, 37]. Therefore, we addressed the question whether *Paracoccidioides* also relied on an SREBP like protein to adapt to hypoxia.

### Identification of SrbA in *Paracoccidioides*

We hypothesized that *Paracoccidioides* hypoxia response could be, in part, regulated by a homologue of the SREBPs, an ancient family of regulators, associated with the hypoxic response in fungi [27, 33–35, 37, 84]. *In silico* analysis using Genbank (http://www.ncbi.nlm.nih.gov/) and *Paracoccidioides* genome databases (http://www.broadinstitute.org/annotation/genome/paracoccidioides_brasiliensis/MultiHome.html) showed that members of the genus *Paracoccidioides*, including the isolate 01, contain homologues of SREBPs. We named the gene *srbA* (*PbsrbA*), and the accession numbers in the *Paracoccidioides* genome database are PAAG_03792, PADG_03295 and PABG_11212 for *Pb*01, *Pb*18 and *Pb*03 strains, respectively.

**Fig 5:**
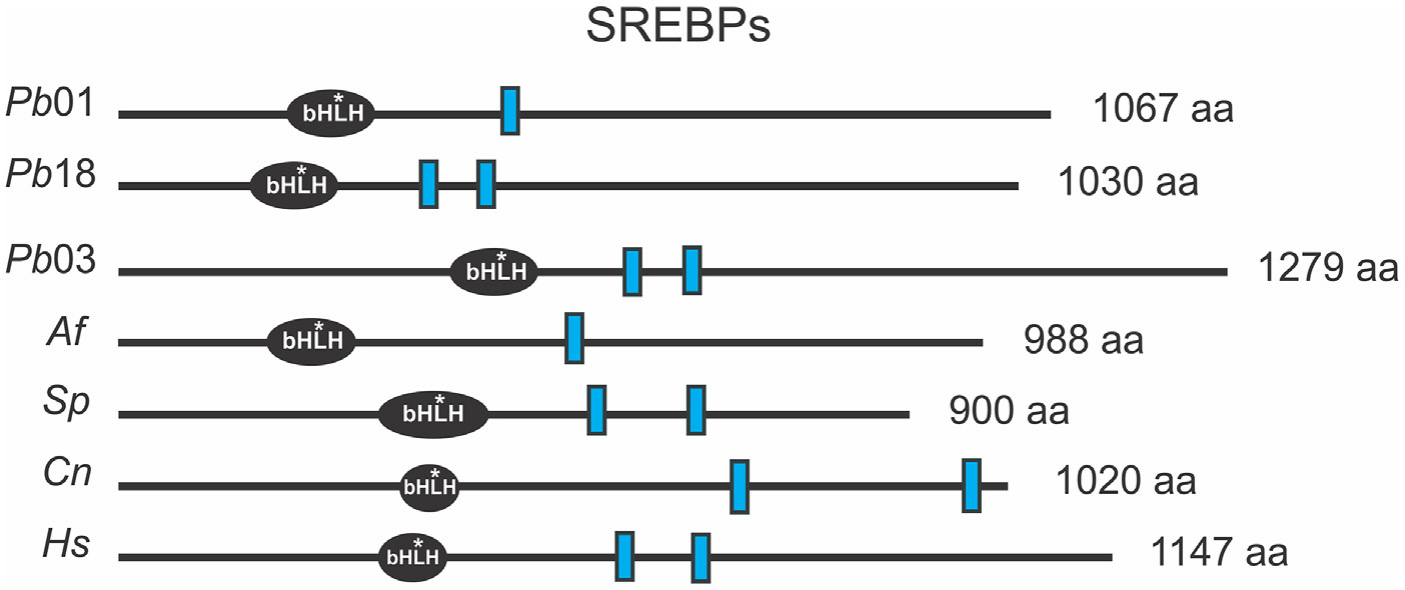
Predicted protein domains for SREBPs component orthologs. The amino acid sequences were obtained from GenBank (http://www.ncbi.nlm.nih.gov/) to *Paracoccidioides Pb*01 (XP_002794199); *Pb*03 (KGY15961); *Pb*18 (XP_010758341); *Aspergillus fumigatus* (XP_749262); *Schizosaccharomyces pombe* (NP_595694); *Cryptococcus neoformans* (XP_567526) and *Homo sapiens* (P36956). The SREBPs bHLH (*basic helix-loop- helix leucine zipper DNA-binding domain*) protein domains and the length of each protein (indicated on the right of each protein in amino acids [aa]) were predicted by SMART tool (http://smart.embl-heidelberg.de) and the transmembrane segments (blue rectangles) were predicted using the Phobius (http://phobius.sbc.su.se/) and SACS MEMSAT2 Prediction softwares (http://www.sacs.ucsf.edu/cgi-bin/memsat.py). *: conserved tyrosine residue specific to the SREBP family of bHLH transcription factors.

The SREBP proteins are basic helix-loop-helix leucine zipper transcription factors with a conserved tyrosine residue, specific to this family. In addition, the SREBP present transmembrane domains, responsible for associating the protein with endoplasmic reticulum (ER). The *Paracoccidioides* spp. *srbA* genes contain those domains (Fig 5 and S1 Text) suggesting that they are an integral membrane protein which requires to be processed to release the N-terminus containing the bHLH DNA binding domain.

In mammals, SREBPs are synthesized as inactive precursors on the endoplasmic reticulum (ER) membrane where they bind to the *S*REBP *c*leavage *a*ctivating *p*rotein (SCAP) which mediates sterol-dependent regulation of SREBP activity. The SCAP protein interacts with another ER-resident protein, named INSIG, and other proteases that cleave into the first transmembrane segment, to release the N-terminal transcription factor SREBP, which translocates to the nucleus and regulates expression of genes required when cholesterol levels are low [27, 28, 38, 85]. In fungi, some differences are detected in the SREBP processing illustrating that, while many aspects of SREBP regulation are conserved across organisms, others are not [45]. In general, the differences are involved with the SREBPs processing for their activation. In *S*. *pombe* and *C*. *neoformans*, SREBPs are regulated in part by proteolysis, although in *S*. *pombe*, this processing is dependent on a Golgi E3 ligase complex, encoded by *dsc* (*d*efective for *S*REBP *c*leavage) genes and not homologues of human proteases, as found in *C*. *neoformans* [86–88]. In *A*. *fumigatus*, the processing is similar to that found in *S*. *pombe* involving the Dsc complex, required for cleavage of SrbA. The hypoxic adaptation and virulence of *A*. *fumigatus* require both, SREBP and its processing mechanism, demonstrating an important mechanism to fungal pathogenesis [37, 45]. *Paracoccidioides* spp., in contrast to *S*. *pombe* and in accordance with *A*. *fumigatus*, does not depict in the genome database homologues for SCAP protein. On the other hand, there is an apparent homolog to the INSIG protein (Table 1). Moreover, the Site-1 and Site-2 proteases homologues were not identified in *Paracoccidioides* spp. genomes, as found in *S*. *pombe* and *A*. *fumigatus* (Table 1). These findings reinforce the relevance of studying activation of SrbA in *Paracoccidioides* spp.

**Table 1:** Conserved SREBP pathway in fungi, including *Paracoccidioides Pb*01, *Pb*03 and *Pb*18.

	Homologues (accession numbers^a^) of SREBP pathway in *Paracoccidioides* complex								
Organism	SREBP^b^	SCAP^c^	INSIG	Site-2 protease	Dsc proteins (*d*efective for *S*REBP *c*leavage)				References
*Paracoccidioides Pb*01	SrbA (XP_002794199)	_	InsA (XP_002796873)	_	DscA (EEH40958.2)	_	DscC (XP_002795148)	DscD (KGQ01980)	In this study.
*Paracoccidioides Pb*03	SrbA (KGY15961)	_	InsA (EEH19174)	_	DscA (EEH21163)	_	DscC (EEH20878)	DscD (KGY15713)	In this study.
*Paracoccidioides Pb*18	SrbA (XP_010758341)	_	InsA (XP_010759317)	_	DscA (XP_010757140)	_	DscC (XP_010757528)	DscD (XP_010759941)	In this study.
*Aspergillus fumigatus*	SrbA (XP_749262)	_	InsA (XP_752057)	_	DscA (XP_752576)	DscB (XP_751662)	DscC (XP_751757)	DscD (XP_754780)	[27, 37, 38, 45].
*Schizosaccharomyces pombe*	Sre 1(NP_595694)	Scp1 (NP_596673)	Ins1 (NP_587813)	_	Dsc1 (NP_595266)	Dsc2 (NP_594090)	Dsc3 (NP_593622)	Dsc4 (NP_594964)	[27, 33, 86, 87].
	Ser 2 (NP_595229)								
*Cryptococcus neoformans*	Sre 1 (XP_567526)	Scp1 (XP_569410)	_	Stp1 (XP_571333)	_	_	_	_	[27, 35, 36, 38, 99].

^a^ NCBI (http://www.ncbi.nlm.nih.gov/) reference sequence accession number (s).

^b^ Sterol regulatory element binding proteins.

^c^ *S*REBP *c*leavage *a*ctivating *p*rotein.

### *Paracoccidioides Pb*01 *srbA* transcript is regulated upon hypoxia

**Fig 6:**
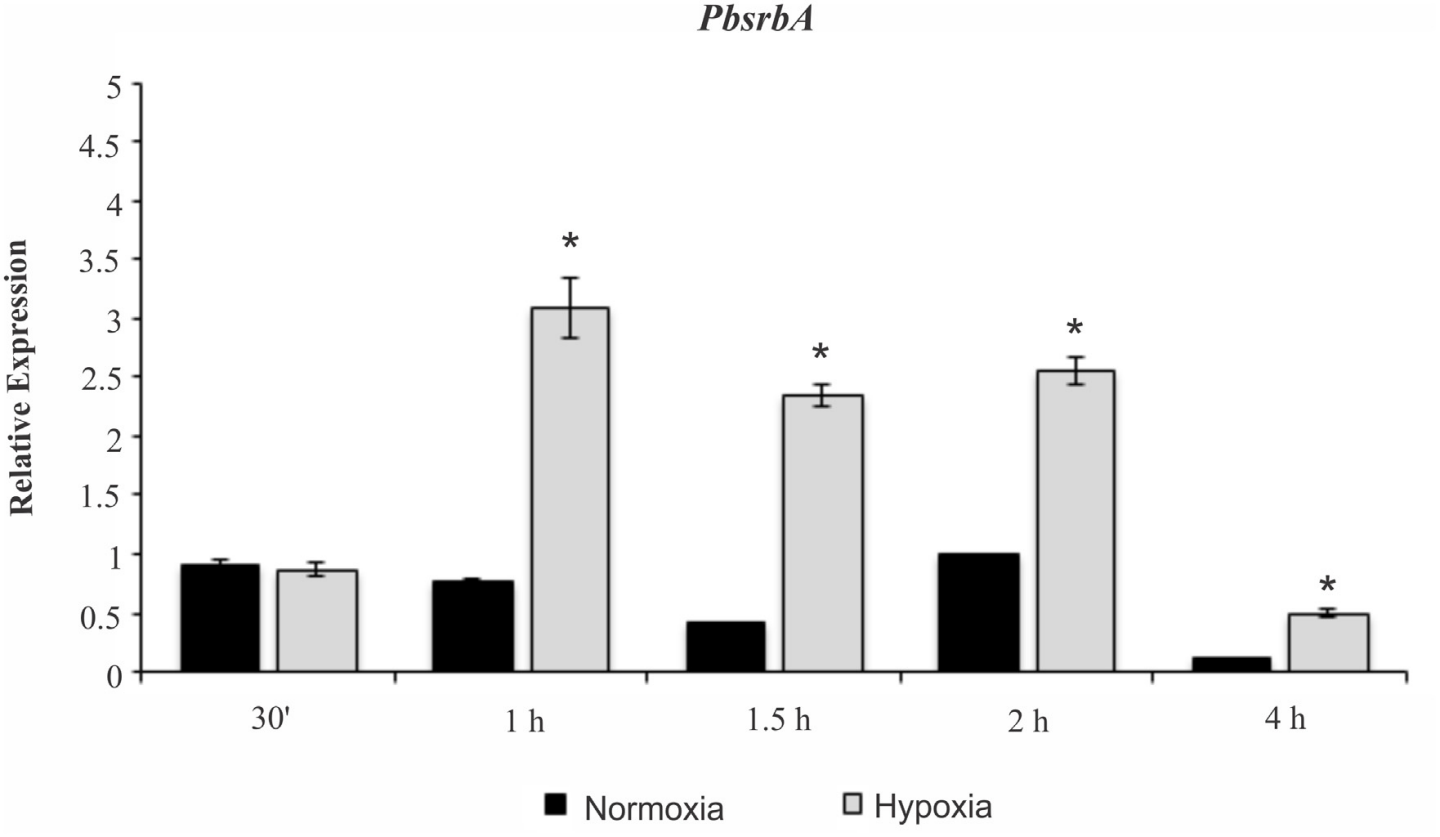
Quantitative RT-PCR revealing *PbsrbA* transcript abundance. *PbsrbA* transcript levels were determined in *Pb*01 yeast cells during hypoxia (1% pO_2_). The cells were incubated at 36°C for 72 h in BHI medium with agitation under normoxia (21% pO_2_) and then were subjected to normoxia and hypoxia from 30 min up to 4 h. The data were normalized using the constitutive gene encoding the alpha tubulin as the endogenous control. Data are also expressed as mean ± SD (represented using error bars) of the three PCR replicates of independent experiments. *, significantly different comparison with normoxia condition, at a P value of ≤ 0.01.

To determine if *PbsrbA* responds to hypoxia, we first examined mRNA levels of the transcript in different oxygen conditions. The fungus significantly increases the levels of *PbsrbA* after 1 h upon hypoxia exposure in comparison to normoxia (Fig 6). These results suggest that *PbsrbA* may be involved in the hypoxia response in *Paracoccidioides* spp. and further analyses were performed to test this hypothesis.

### *Paracoccidioides Pb*01 *srbA* is required for hypoxia adaptation, resistance to azoles and iron acquisition

There are a reduced number of works relating the functional analysis of genes in *Paracoccidioides* in the last six years because the achievement of viable and stable mutants of *Paracoccidioides* spp. is a hard task [11, 13, 89–94]. This parsimony in functional analysis surely reflects the complexity of those studies in the genus *Paracoccidioides*, as well as in other pathogenic fungi. Due these limitations in molecular genetic analyses available in *Paracoccidioides*, we utilized a heterologous genetics approach to test our hypothesis.

In order to test whether *Paracoccidioides srbA* was able to replace the *A*. *fumigatus* SrbA function, we introduced *Paracoccidioides srbA* (*PbsrbA*) under control of the *gpdA* (glyceraldehyde-3-phosphate dehydrogenase) promoter from *A*. *nidulans* into a previously characterized *srbA* null mutant strain *A*. *fumigatus* (*ΔsrbA*) [45]. Ectopic introduction of the *Paracoccidioides srbA* gene (*PbsrbA*) into *ΔsrbA* allowed us to attribute all resulting phenotypes specifically to the absence of *srbA* in *A*. *fumigatus* [37, 45]. Colonies were exposed to low oxygen growth condition (1% pO_2_) to randomized screening (S1 Fig) and confirmation of the strain genotype was done with Southern blot and PCR analyses (S2 Fig). A total of one and two copies of the *PbsrbA* and *pyrG* gene was observed in Rec1 (reconstituted strain 1) and Rec2 (reconstituted strain 2), respectively. The detected high band on *pyrG* Southern blot results (around 5 kb) is an unspecific cross-reactive detection because the probe is able to recognize the non-functional *pyrG* used to knockout the *srbA* gene in *A*. *fumigatus* genome [45] (S2A Fig).

We next confirmed the *PbsrbA* genome integration using conventional PCR, using primers that amplify the *PbsrbA* sequence including the *AngpdA* promoter (S2B Fig). In addition, the *PbsrbA* transcript and protein expression were assessed (S3 Fig). As expected, the *PbsrbA* transcript was expressed only in the reconstituted strains (Rec1 and Rec2), increasing when the fungus was submitted to hypoxia (S3A Fig). In agreement, the *AfsrbA* transcript was not detected in the reconstituted strains. The transcript to *AfsrbA* was also analyzed and the results are consistent with previously published data and reinforce the obtained data with *PbsrbA*. Using quantitative real time PCR, we observed that the transcript to *AfsrbA* was expressed only in the wild type strain, increasing when the fungus faced hypoxia (S3A Fig). In addition, at the protein level, the western blotting analysis, using a polyclonal antibody against *A*. *fumigatus* SrbA amino acids 1–275, indicates that *PbsrbA* is expressed in the reconstituted strain (Rec1) (S3B Fig). The *A*. *fumigatus* SrbA protein was also detected in the wild-type strain showing the SrbA precursor and N-terminal cleavage protein [45].

**Fig 7:**
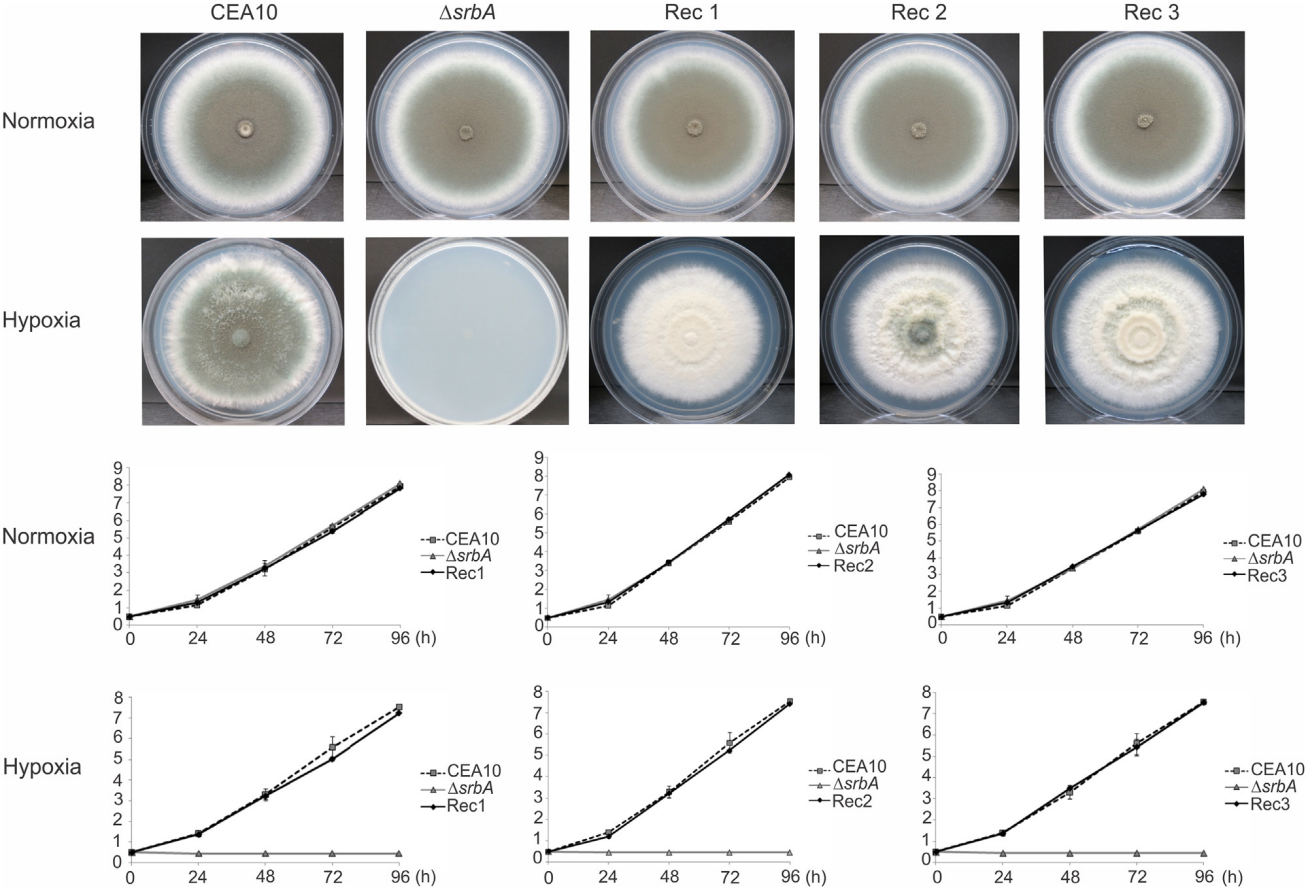
*PbsrbA* is required for hyphal growth under hypoxic conditions. The *A*. *fumigatus* reconstituted strains (*PbsrbA*), wild type (CEA10) and *ΔsrbA* were plated on GMM plates and incubated at 37°C under normoxia and hypoxia. The diameter of the colony was measured over 96 h every 24 h and are expressed in inches (cm). Under normoxia, no significant difference in growth speed and colony size could be observed (P ≤ 0.01) except less conidiation in the reconstituted strains (upper panel). In contrast, under hypoxic conditions only the mutant strain (*ΔsrbA*) did not demonstrate any detectable growth. The reconstituted strains 1, 2 and 3 (Rec1, 2 and 3) and the wild type (CEA10) showed comparable growth; P ≤ 0.01 (lower panel).

In order to analyse the growth of the reconstituted strains exposed to hypoxia, we measured the colony diameter of each strain every 24 h (Fig 7). As previously described, the *A*. *fumigatus srbA* null mutant strain does not growth under hypoxia [37]. However, the *PbsrbA* reconstituted strains were able to restore the null mutant hyphal growth under hypoxia (Fig 7). This result indicates that *Paracoccidioides* has a functional SrbA protein that can rapidly promote adaptation to hypoxic microenvironments.

**Fig 8:**
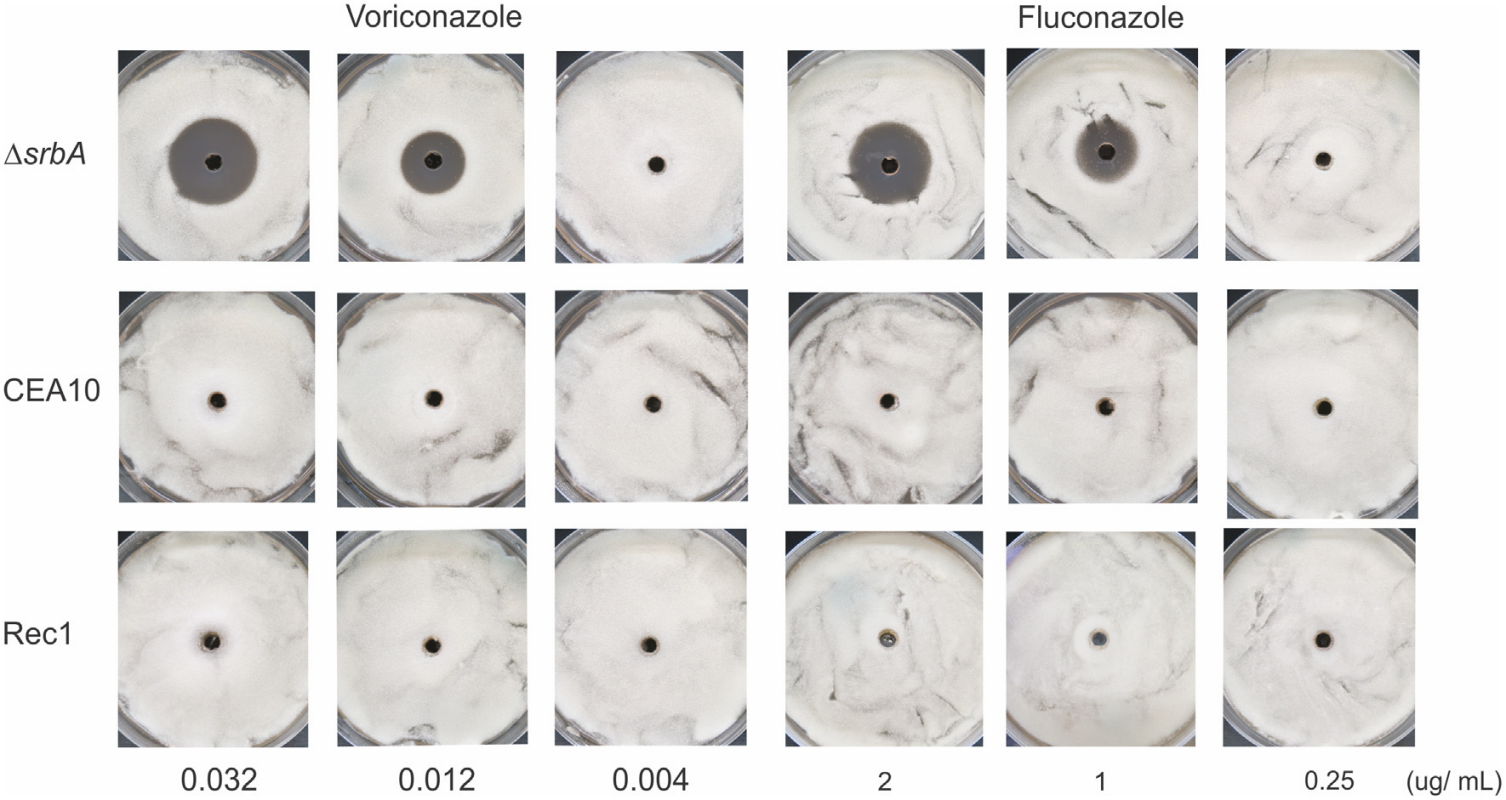
Analysis of resistance of the wild type (CEA10), *ΔsrbA* and the Rec1 strains of *A*. *fumigatus* to fluconazole and voriconaloze. The susceptibility of the Rec1 (*PbsrbA*), wild type (CEA10) and *ΔsrbA* of *A*. *fumigatus* were analyzed to both drugs, fluconazole and voriconazole. The concentrations of each drug were choose based on previously published data [37]. The fluconazole and voriconazol have no effect on wild type and Rec 1 strains showing that *PbsrbA* could restore the phenotype of wild type. In contrast, the mutant was susceptible for both drugs, from 1 and 0.012 ug/ ml of fluconazole and voriconazol, respectively.

Previous studies showed that the *A*. *fumigatus* SrbA protein coordinates iron and ergosterol homeostasis to mediate triazole drug and hypoxia responses [37, 95]. The *A*. *fumigatus* SREBP is a key positive regulator of iron homeostasis, particularly related to iron acquisition, which is essential for adaptation to hypoxia and low iron microenvironments [95]. Iron homeostasis has been characterized in *Paracoccidioides* [11, 12, 96–98] and the elucidation of additional molecules involved in this process can be relevant in the understanding of fungus pathogenesis. In this sense, our purpose was firstly attempted to screen *PbsrbA* reconstituted strain susceptibility to antifungals drugs using ranges of azoles concentrations [37] (Fig 8). The results showed that *PbsrbA* restores the failed growth of the mutant and suggest its participation in mechanisms of resistance to azoles. Previous studies in *S*. *pombe*, *C*. *neoformans*, and *A*. *fumigatus* confirmed that fungal SREBPs are key regulators of ergosterol biosynthesis [33, 36, 39, 77]. In *A*. *fumigatus*, the SrbA protein is involved, even in part, in regulation of the expression of several ergosterol biosynthesis genes [37, 95]. Taken together, the results suggest that *PbsrbA* can also be involved in these mechanisms because transcript to *Pberg3* involved in ergosterol biosynthesis production, is also regulated in *Paracoccidioides Pb*01 submitted to hypoxia (Fig 4). Possibly, the fungus increases the expression of genes related to ergosterol biosynthesis, in order to compensate the reduction in ergosterol production in low oxygen, as discussed before in Fig 3.

**Fig 9:**
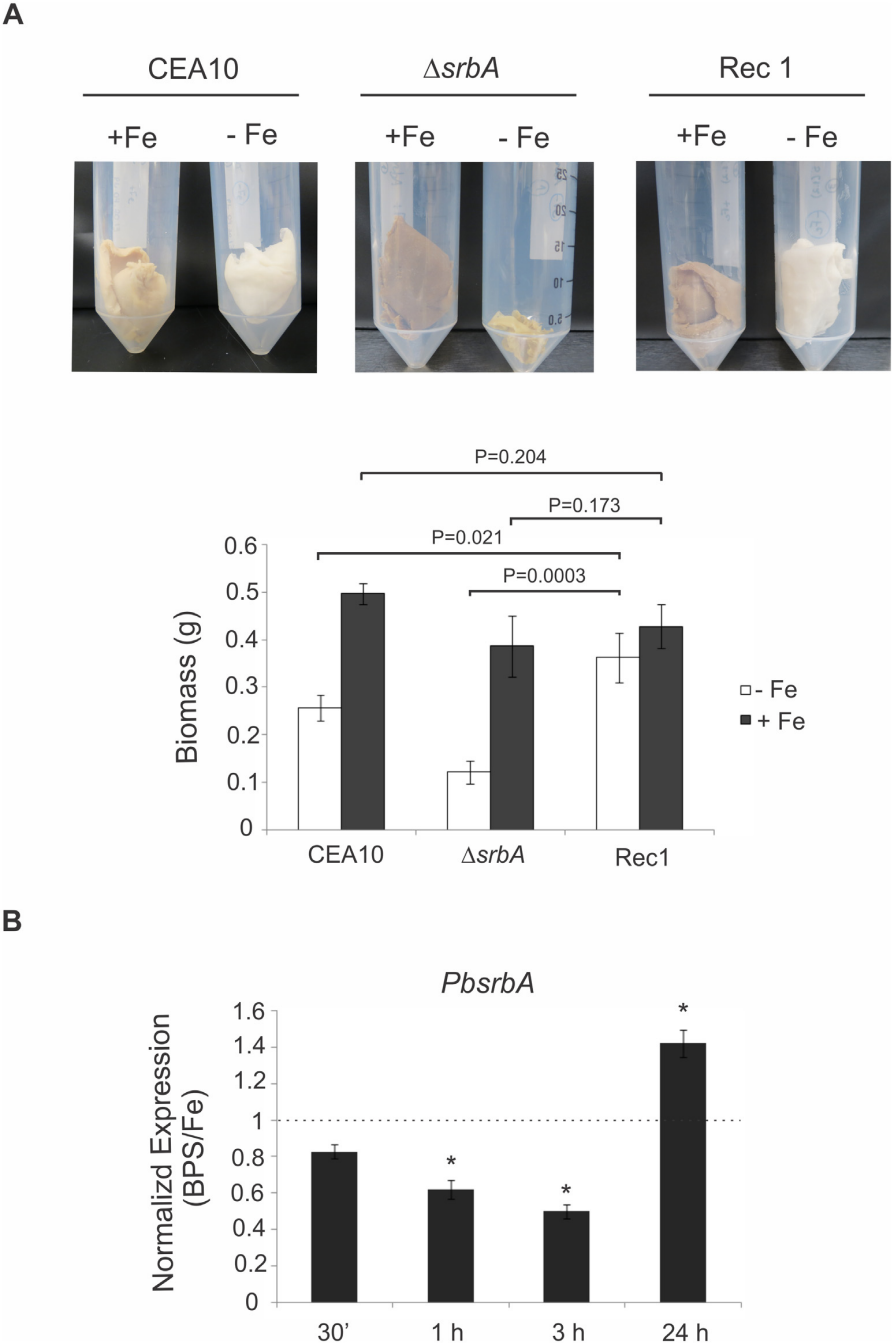
*PbsrbA* restores the deficient growth of the *A*. *fumigatus srbA* mutant under iron starvation. **(A)** A total of 10^8^ conidia of each wild type (CEA10), *ΔsrbA* and Rec1 (*PbsrbA*) strains of *A*. *fumigatus* in 50 ml liquid GMM was monitored after 48 h of growth at 37°C under iron starvation (-Fe) and iron availability (+Fe, 30 μM). The biomass production (dry mass) was compared among them, and expressed in grams (g). The data represent the mean ± standard deviation of biological triplicates. The difference between reconstituted strain and mutant/ wild-type strains was statistically significant during -Fe but not +Fe (t-test; P ≤ 0.05). **(B)** The analysis of transcripts abundance of *PbsrbA* was performed using qRT-PCR when yeast cells of *Paracoccidioides Pb*01 were submitted to iron sufficiency (30 μM) and deprivation (iron chelator bathophenanthrolinedisulfonate, BPS [50 μM]) culture media. Total RNAs were extracted and treated with DNAse. The cDNAs molecules were synthesized and qRT-PCR performed. The values that were plotted on the bar graph were normalized against the expression data that were obtained from the no iron addition condition (fold change). The data are expressed as the mean ± standard deviation of the triplicates. *statistically significant data as determined by Student’s t-test (P ≤ 0.01).

Regarding iron homeostasis, previous studies showed that the initial responses to hypoxia in *A*. *fumigatus* involve transcriptional induction of genes involved in iron acquisition. The null mutant strain to *srbA* (Δ*srbA*) has reduced growth under iron starvation in liquid medium because it coordinates responses to iron and oxygen depletion [39, 95]. Here the *PbsrbA* reconstituted strain 1 (Rec1) was significantly able to restore the defective growth phenotype of the mutant (Fig 9A). In fact, the transcript to *PbsrbA* is up-regulated in *Paracoccidioides* sp. grown upon iron deprivation, mainly after 24 h of incubation (Fig 9B). Even in part, this gene could be important in the mechanisms to compensate the effect of iron depletion in *Paracoccidioides* sp. yeast cells.

Altogether, the results show that the roles of *srbA* are also conserved in *Paracoccidioides* especially those related to hypoxia, susceptibility to the azoles and iron deprivation responses. Even partially, the *Pb*01 SREBP was able to restore the mutant phenotypes similarly to wild type strain. In this way, SREBP is a relevant molecule to compensate the effects of hypoxia in *A*. *fumigatus* and in *Paracoccidioides*.

In conclusion, the hypoxia response of *Paracoccidioides* spp. was largely unknown. In this study, we used a large-scale proteomic approach and a detailed functional characterization of the homologue to the most important molecule involved in hypoxia responses in other fungi, the SREBP protein. Our results show that *Paracoccidioides* modulates several metabolic pathways in order to compensate for hypoxia stress and importantly it has a functional SREBP homologue, the SrbA protein, which could be involved in regulation of the majority of the hypoxia responses in this pathogen. Taken into account that hypoxia is an important condition faced by pathogens during infection, this characterization becomes relevant in the context of *Paracoccidioides* spp. pathogenesis and warrants further investigation.

## Supporting Information

S1 Fig Screening of the *Aspergillus fumigatus* transformants containing *PbsrbA* in the genome. In order to select positive colonies, the *A*. *fumigatus* transformants were submitted to hypoxia (1% pO_2_) and normoxia (21% pO_2_). The growth indicates possible positive colonies in which *PbsrbA* was inserted on *A*. *fumigatus* genome. WT: wild type; Rec: reconstituted strains with *PbsrbA* gene; *ΔsrbA*: null mutant for *A*. *fumigatus srbA* gene.
(TIF)

S2 Fig Confirmation of the ectopic reconstitution of the *PbsrbA* in the *A*. *fumigatus srbA* null mutant using Southern blot and conventional PCR. **(A)** Southern blot analysis of *A*. *fumigatus* wild type (CEA10), *ΔsrbA* and *PbsrbA* reconstituted strains (Rec1 and Rec2). Genomic DNA from the respective strains was isolated and digested overnight with *Hind*III and *Eco*RI restriction enzymes. Probes to *gpdA* and *pyrG* probes, were used. Expected hybridization patterns were observed to both probes. The *gpdA* promoter was detected in the reconstituted strains and not in wild type and *ΔsrbA* from *A*. *fumigatus*. The total of one and two copies of the *PbsrbA* was observed in reconstituted strains 1 and 2 (Rec1 and Rec2), respectively. Similarly, a total of one and two copies of the *pyrG* gene was observed in Rec1 and Rec2 strains, respectively. Besides Rec1 and Rec2, a high band of the *pyrG* gene (around 5 kb) was observed in strains, except in wild type. The detected high band is an unspecific cross-reactive detection because the probe is able to recognize the non-functional *pyrG* used to knockout the *srbA* gene in *A*. *fumigatus* genome [45]. **(B)** Primers were used to amplify *PbsrbA* and *gpdA* DNA fragments to further confirmations. Seven reconstituted strains were chosen as positive to insertion. Primers A and B (S3 Table) amplify a fragment including the *gpdA* promoter fused to *PbsrbA* (4.8 kb) and the primers A and C (S3 Table) only the *gpdA* fragment (1.5 kb), as indicated in the scheme below. A positive control indicates the same PCR reaction using the *A*. *nidulans* DNA genomic as template.
(TIF)

S3 Fig The reconstituted *A*. *fumigatus* strain is able to produce *PbsrbA* transcript and protein. **(A)** The *PbsrbA* transcript abundance was assessed using qRT-PCR in *A*. *fumigatus* wild type (CEA10), *ΔsrbA* (KO) and *PbsrbA* reconstituted strains 1 and 2 (Rec1 and Rec2). Germlings were grown from conidia at 23°C for 18 h. The strains were submitted to hypoxia (1% oxygen and 5% CO_2_) for 2 and 4 h in liquid GMM with agitation. Total RNA was extracted and treated with DNase. Equal amounts of DNase-treated RNA (500 ng) were reverse transcribed and used for qRT-PCR. Primers are depicted in S3 Table. Error bars represent the standard deviations of the means for three biological replicates. The data were normalized using the *A*. *fumigatus tefA* as endogenous gene and they were plotted as a relative expression level of each gene to each condition. **(B)** Immunoblot was performed using protein extracts from *A*. *fumigatus* wild type (CEA10), *ΔsrbA* and *PbsrbA* reconstituted strain 1 (Rec 1). Germlings were grown from conidia at 23°C for 18 h in baffle flasks and were submitted to normoxia (N) or hypoxia (H) for 1 h at 37°C. On the right of the blot, * indicates the precursor *Pb*SrbA protein band and # indicates the processed N-terminus protein band. The antibody recognizes the N-terminal *A*. *fumigatus* SrbA protein, which express the first 425 amino acids of full-length SrbA. To *A*. *fumigatus*, the strongest band on immunoblots under normoxia was observed at about 120 kDa corresponding a precursor protein and the N-terminal cleavaged protein of SrbA, of ~60 kDa (containing the bHLH domain) was also found on immunoblots from the wild type [45].
(TIF)

S1 Text Sequences and ClustalX2 alignment between *Paracoccidioides* SrbA and its orthologs in *Aspergillus fumigatus*, *Schizosaccharomyces pombe*, *Cryptococcus neoformans* and *Homo sapiens* (SREBP-1). The portion of the predicted DNA-binding domain are depicted yellow colour with a conserved tyrosine residue (green colour) specific to the SREBP family of bHLH transcription factors.
(DOCX)

S1 Table Up-regulated proteins of *Paracoccidioides* (*Pb*01) yeast cells under oxygen deprivation for 12 and 24 h detected by NanoUPLC_MS^E^ analysis. (DOCX)

S2 Table Down-regulated proteins of *Paracoccidioides* (*Pb*01) yeast cells under oxygen deprivation for 12 and 24 h detected by NanoUPLC_MS^E^ analysis. (DOCX)

S3 Table Oligonucleotides used in this study. (DOCX)
