# Supplementary figures and images for: Characterization of the Paracoccidioides Hypoxia Response Reveals New Insights into Pathogenesis Mechanisms of This Important Human Pathogenic Fungus
Source: PLoS Negl Trop Dis. 2015 Dec 10;9(12):e0004282. doi: 10.1371/journal.pntd.0004282 (PMC4686304; doi:10.1371/journal.pntd.0004282)

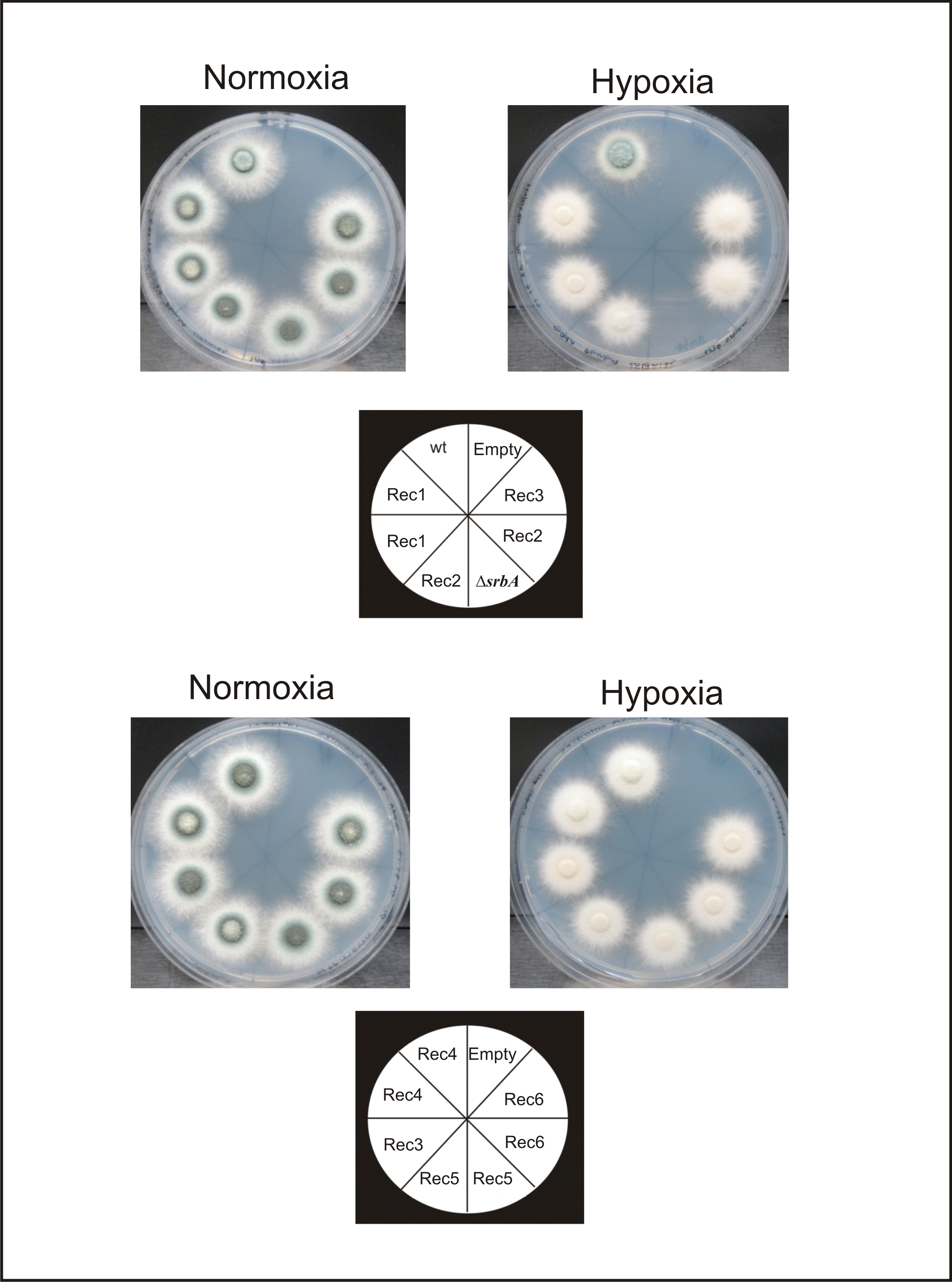

Supplement: S1 Fig — In order to select positive colonies, the A. fumigatus transformants were submitted to hypoxia (1% pO2) and normoxia (21% pO2). The growth indicates possible positive colonies in which PbsrbA was inserted on A. fumigatus genome. WT: wild type; Rec: reconstituted strains with PbsrbA gene; ΔsrbA: null mutant for A. fumigatus srbA gene. (TIF) [file pntd.0004282.s001.tif]

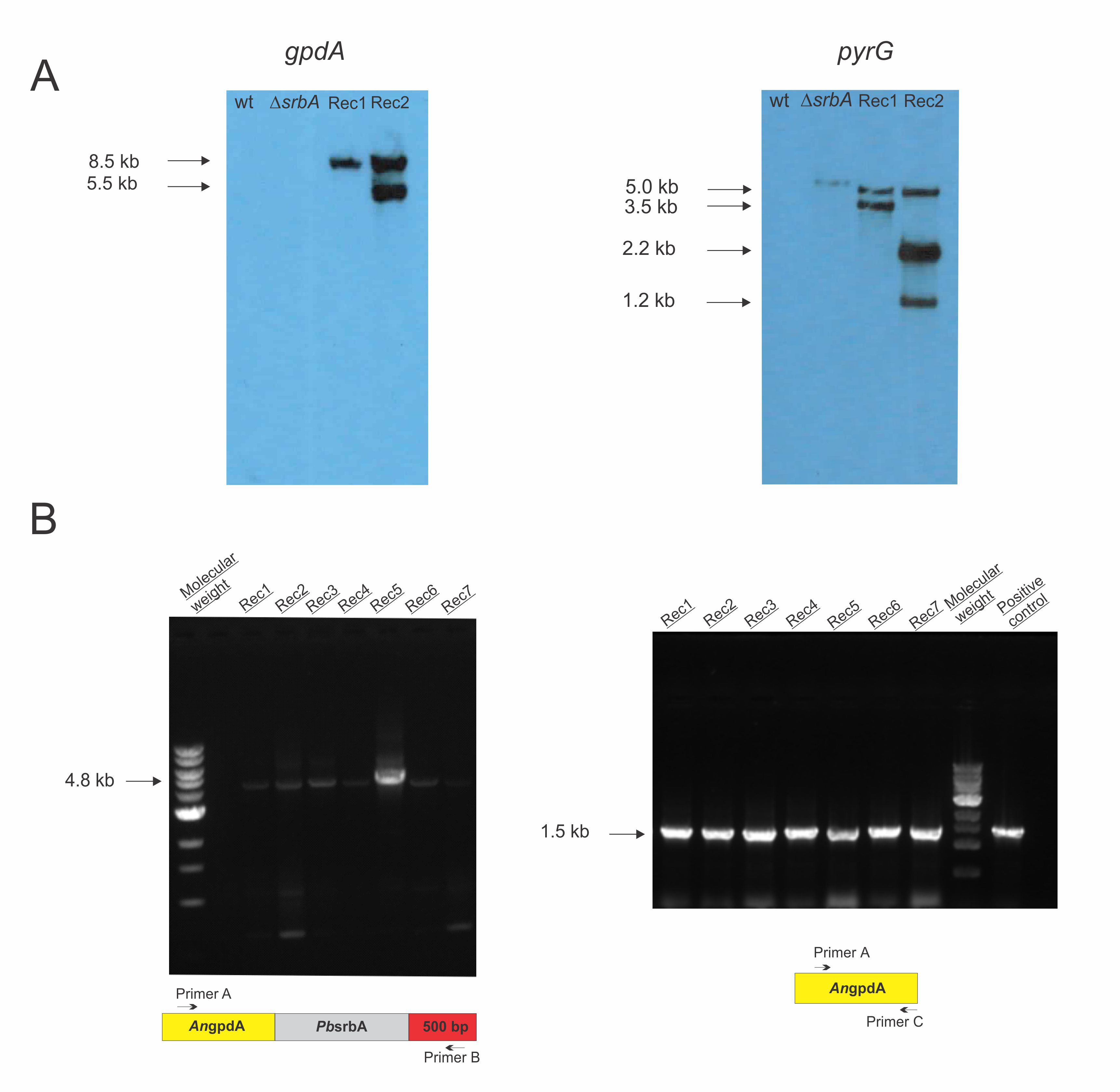

Supplement: S2 Fig — (A) Southern blot analysis of A. fumigatus wild type (CEA10), ΔsrbA and PbsrbA reconstituted strains (Rec1 and Rec2). Genomic DNA from the respective strains was isolated and digested overnight with HindIII and EcoRI restriction enzymes. Probes to gpdA and pyrG probes, were used. Expected hybridization patterns were observed to both probes. The gpdA promoter was detected in the reconstituted strains and not in wild type and ΔsrbA from A. fumigatus. The total of one and two copies of the PbsrbA was observed in reconstituted strains 1 and 2 (Rec1 and Rec2), respectively. Similarly, a total of one and two copies of the pyrG gene was observed in Rec1 and Rec2 strains, respectively. Besides Rec1 and Rec2, a high band of the pyrG gene (around 5 kb) was observed in strains, except in wild type. The detected high band is an unspecific cross-reactive detection because the probe is able to recognize the non-functional pyrG used to knockout the srbA gene in A. fumigatus genome [45]. (B) Primers were used to amplify PbsrbA and gpdA DNA fragments to further confirmations. Seven reconstituted strains were chosen as positive to insertion. Primers A and B (S3 Table) amplify a fragment including the gpdA promoter fused to PbsrbA (4.8 kb) and the primers A and C (S3 Table) only the gpdA fragment (1.5 kb), as indicated in the scheme below. A positive control indicates the same PCR reaction using the A. nidulans DNA genomic as template. (TIF) [file pntd.0004282.s002.tif]
