# Supplementary material for: Characterization of the Paracoccidioides Hypoxia Response Reveals New Insights into Pathogenesis Mechanisms of This Important Human Pathogenic Fungus
Source: PLoS Negl Trop Dis. 2015 Dec 10;9(12):e0004282. doi: 10.1371/journal.pntd.0004282 (PMC4686304; doi:10.1371/journal.pntd.0004282)
Supplement: S1 Text — The portion of the predicted DNA-binding domain are depicted yellow colour with a conserved tyrosine residue (green colour) specific to the SREBP family of bHLH transcription factors. (DOCX) [file pntd.0004282.s004.docx]

The amino acid sequences were obtained from GenBank (<http://www.ncbi.nlm.nih.gov/>) to *Paracoccidioides Pb*01 (XP_002794199); *Pb*18 ([XP_010758341](http://www.ncbi.nlm.nih.gov/protein/734676124?report=genbank&log$=prottop&blast_rank=3&RID=TWU2A7J9014)); *Pb*03 (KGY15961) ; *Aspergillus fumigatus* (XP_749262); *Schizosaccharomyces pombe* (NP_595694); *Cryptococcus neoformans* (XP_567526) and *Homo sapiens* SREBP-1 (P36956). The predicted DNA-binding domain and the conserved tyrosine residue are shown in yellow and green colours, respectively. The conserved tyrosine residue is specific to the SREBP family of bHLH transcription factors.

>*Pb*01 (XP_002794199)

MSFSQSPLRVSMSPSTDISSNNSPRQALTSDWSQWMQWDGFESDDISGHTDLFSLPPKTEIKTIDTNQTL

PLKSFDPPPPVDSAFTFTAGSLPICETISPQEPSCVSDTSPAPSDFFDSALSPGVADGRWNKSGATIPLP

PDNNRSKQPLEGKKCSLPSAPLPSFPITDRKRKTRPDEPPGSAGADDSPVSSSKNLPSKKRSHNVIEKRY

RANLNEKIAELRDSVPSLRALAKQKSGISCQAVQDDDEIVSSSNKLNKASILSKATEYIRHLELRNKRLE

EENVALKNRLRQLEKVQEQNVVNYASTSGSVSSPDAYTVSTDSGAGSSPGVFSQNEDTSPESSPNPLYPP

EGLIKVPEYFKRMRMTGPQPHYADSFIQRPNANTTYTSSERKGTIIPNKFMLGTLAALMIVGGFESQKSE

SSEKGLLGIPLQFAGEIHRFCQTHLCFPTTTSWQIRALSHFALTSLVVVGAAFAVFLYLFNSAPRRAKHP

AKSTTGSRNPAAVSPIEFRREAWLTSIQTVWVPSHNFFPEWFAVTWRCMEYVLSCILGWKLYSWLTGITE

EDETARAKAWDIAIDAQLTGGDPEVSKSRMVLTIFAAGTLPSTPARVMLKALHCRLLLWMVGSRGSWAFR

LSNHIAFLLASYQWEIARNLQKSLPKDHDDSLPDHLATLLDANIDDVMTDAIIQRASNMAWNRCTQEATY

GEDAMLDIVVEDTAVRSPLDALAAWWSSRALQNALIHCLDVDTAEPSAQRTKAFEQGLTLALNVAPIASA

AYTRAAVIKAVFFDEDRVANINTTLAALPRSKSQSAISSTNFLDSSVPPSARAEISIAVRCAMIAAILRG

QVDTNPVTSPQLTLENAITLFNILPVDPVELTLLGFASLYHLLHIVAAEPWMVLGSSLSSPISSSSSDTS

SARQDGESSDIDCHPLPTPDLARIASGLIYWVRNAYNPVSSGFDMELAEKAVKGCVDVCQSVGIHIEAAE

TKWEGHLVDNSAQGSQITQSTASEGRTNHSGDGGWEESQQNVENVRRESLESTDTGYGSLSQEDERADNK

EKKITATQRVEGVYLTN

>*Pb*18 ([XP_010758341](http://www.ncbi.nlm.nih.gov/protein/734676124?report=genbank&log$=prottop&blast_rank=3&RID=TWU2A7J9014)**)**

MSSSQSPLRVSMSPSTDFSSNNSPPQALTSDWSQWMHLPPKTEIKTIDTNHTLPLKCFDPPPPVDSAFTFTAGSLPLCETVSPQEPSCVSDTSPAPSDFFDSALSPGVADGRWNNSGATIPLPADNHRTKPPEGKKCSLHPSAPLPSFPITDRKRKTRPDEPPGSAGAGDSPVTSSKNLPSKKRSHNVIEKRYRANLNEKIAELRDSVPSLRALAKQKSGISCQAVQDDDEIVSSSNKLNKASILSKATEYIRHLELRNKRLEEENVALKNRLRQLEKVQEQSVVNYASMSGSVSSPDAYTVSTDSGTGSSPGVFSQNDDPSPESSPNPLYPPEGLIKVPEYFKRMRMTGPQPHYADSFIQRPNTTTTTYTSSERKRTFIPNKFMLGTLAALMIVGGFESHKSESSEKGLLGLPLQFAGEIHRFCQTHLCFPTTTSWQIRALSHFALTSLVVVGAAFAVFLYLFNSAPRRAKHPAKSTTGSRNPAAVSPIEFRREAWLTSIQTVWVPSHNFFPEWFAVTWRCMEYVLSCILGWKLYSWLTGITEEDETARAKAWDIAIDAQLTGGDPEVSKSRMVLTIFAAGTLPSTPARVMLKALHCRLLLWMVGSRGSWAFRLSNHIAFLLASYQWEIARNLQKSLPKDHDDSLPDHLATLLDANIDDVMTDAIIQRASNMAWNRCTQEATYGEDAMLDIVVEDTAVRSPLDALAAWWSSRALQNALIHCLDVDTVEPSAQRTKAFEQGLTLALNVAPIASAAYTRAAVIKAVFFDEDRVANINTTLAALPRSKSQPAISSTNFLDSSVPPSARAEISIAVRCAMIAAILRGQVDTNPVTSPQLTLENAITLFNILPVDPVELTLLGFASLYHLLHVVAAEPWMVLGSSLSSPISSSSSDTSSARQDGESSDTDGHTLPTPDLARIASGLIYWVRNAYNPVSSGFDMELVEKAVKGCVDVCQSVGIPIEAAEIQWEGHLVNNSAQGSQITQSTASEGRTSHSGDGGWEESQQNVENVRRESLESTDTGYGSLSQEDERADNKEIKITATQRVEGVLSHQLGSSR

>*Pb*03 (KGY15961)

MRRKPPATKDRARSNEIKIIDCQTAAATITQPAHYPAQTPSTTPSTTPSKYTASTQQVPPASTPSTLPTQHLVPSAATPPYPIPSTPRTQYPATPRTHQRPTRNPPNRPPPNRPCPRPRTGRPPAARDKELRRPTSKGERRRTKDQRRSLGGAGQSGPIFTPTASPPPLTRDEETDRNRSFIVRLYALISKREPAFYLLICLFVIAMSSSQSPLRVSMSPSTDFSSNNSPPQALTSDWSQWMQWDGFESDDISGHTDLFSLPPKTEIKTIDTNHTLPLKCFDPPPPVDSAFTFTAGSLPLCETVSPQEPSCVSDTSPAPSDFFDSALSPGVADGRWNNSGATIPLPADNHRTKPPEGKKCSLHPSAPLPSFPITDRKRKTRPDEPPGSAGAGYSPVTSSKNLPSKKRSHNVIEKRYRANLNEKIAELRDSVPSLRALAKQKSGISCQAVQDDDEIVSSSNKLNKASILSKATEYIRHLELRNKRLEEENVALKNRLRQLEKVQEQSVVNYASMSGSVSSPDAYTVSTDSGTGSSPGVFSQNDDPSPESSPNPLYPPEGLIKVPEYFKRMRMTGPQPHYADSFIQRPNTTTTTYTSSERKRTFIPNKFMLGTLAALMIVGGFESHKSESSEKGLLGLPLQFAGEIHRFCQTHLCFPTTTSWQIRALSHFALTSLVVVGAAFAVFLYLFNSAPRRAKHPAKSTTGSRNPAAVSPIEFRREAWLTSIQTVWVPSHNFFPEWFAVTWRCMEYVLSCILGWKLYSWLTGITEEDETARAKAWDIAIDAQLTGGDPEVSKSRMVLTIFAAGTLPSTPARVMLKALHCRLLLWMVGSRGSWAFRLSNHIAFLLASYQWEIARNLQKSLPKDHDDSLPDHLATLLDANIDDVMTDAIIQRASNMAWNRCTQEATYGEDAMLDIVVEDTAVRSPLDALAAWWSSRALQNALIHCLDVDTVEPSAQRTKAFEQGLTLALNVAPIASAAYTRAAVIKAVFFDEDRVANINTTLAALPRSKSQPAISSTNFLDSSVPPSARAEISIAVRCAMIAAILRGQVDTNPVTSPQLTLENAITLFNILPVDPVELTLLGFASLYHLLHVVAAEPWMVLGSSLSSPISSSSSDTSSARQDGESSDTDGHTLPTPDLARIASGLIYWVRNAYNPVSSGFDMELVEKAVKGCVDVCQSVGIPIEAAEIQWEGHLVNNSAQGSQITQSTASEGRTSHSGDGGWEESQQNVENVRRESLESTDTGYGSLSQEDERADNKEIKITATQRVEGVLSHQLGSSG

>*Afumigatus* (XP_749262**)**

MSTPGIGGDFQLFSPLESTRRISQGNSLSVDQSSTDVASQDWTQWMRWDDEQAFPETANASPSSPFDLAF

ISPSASSGREASDAMHKDFSPDISLDFKSPSLGFFPGGDLNTNVSPQPDHVGAGSLSVHSNSPLSSIGAS

RKRKTGSDDDGSTMTSMFKAKQAPSKKRAHNVIEKRYRANLNEKIAELRDSVPSLRASYKQANGNSGDDD

DDGVTSASKLNKASILSKATEYIRHLEIRNKRLEEENTALKIRLRQLDKAADQIVTSAASVSSPSDCTVS

TESGASSSPSVFSHAEDVPSDHSPTSSHPPEGLIKVPDAWKRMRAAGSNESPYSQSYIQYKKTDSHSSQS

GGGRMRSHLPNKYMLGALAGLMVLEGLGTEKKTESTAKGLLAVPLNLLNRVQLPSEVYSSAAFQYFWSSW

HARAISHFLMLAILVVGSAFIVFVYLFNSDPRRQYSASKVAPDVTLSSCNFRRQAWLTSIQRVGVPRHRF

FHEWYVVTSRCFEYVLRCLLGWKLYSLVTGVTEEDEKGRVKTWDIAIDAQLAGGDAEISKSRLVLTIFAA

GTLPRSPMRMMLKALHCRILMWRVGEPGSWTFNVSNDVARSLARYQWDLARKMNAALPKDHPDSLPSHLA

TLLKIDCDDVMIDTIIQRAANLTWNRPTQEGTDDDEALLDVVEEDPAIQSSLDALAAWWSSHLLQGALLR

YFEASSGGPDAKKSRNVFKSKIKLALDVAPQPSAAHTRALVMMAVFFERDRVANIGSVLAALPKEKGKNK

QNQASNFLDSSLPISVREEISTAVRCAMIAAIFNARATGDTSLPATFTVEKAIHWFNRLPLDPVELTLLE

FAAVYHLLHILASDIDYLASSDSSAPPSPMSKASDMLSSSSDDGEDGASQRNNNIIPHPMPNLGRVASEL

IYWARNAYNPAFYGFTSNLVKVIETECTSLCQTAGVHVADYSCVQEEKSKAKQAIDSKRRFAGGNEEASD

NLLLSDES

>*Spombe* (NP_595694)

MQSSIPSVSVSVASPAMETPTKASPDSKSPNSVGAIPSSSPLASSTKASTSTPFVENCSNLLCDLASIVE

DSPPTLTNTSLSPHSFSLSDMESSMSNWLNPFAFDNTMNSAPPLFTSTNMGSPNSLENSTNPLLSNCGSP

NSFQNETFTGPSLNEFDADDKIQRQMKILHSVDTIDPSTVQNYPSADMSNPEVKLKTEEIITPMDTTCKP

EPSAKKIKLSPSSEDSCSIPETLPFSAPKSRGSLSPSETPDFVAGPGGKPKKTAHNMIEKRYRTNLNDRI

CELRDAVPSLRAAAALRCGNSLDDEDLGGLTPARKLNKGTILAKATEYIRHLEAKNKELQKTNKQLSDRL

AFYEDPSMAPPSNDTRAVNSVNVVSSSDYSVHQSSRPNLTQRAFTSPTLNTMGRTALNGMVGLGLFNYFG

NDSSQSVYGLFALPPFLMSPFTGTVLFNMLKIGVVLLGLFYLLHDNSLFKGFKGEKKSKVSTRSSMSPSS

ILFRKTVFEKYCLLDHSTSTISLFFGLLIFTLKSAYGYLTHRLSALYTSSENWVYSEQQLAEVRNMEKLL

DAQLMGGDAKVDRLRLLMVFASSFSLPPSSHTCALQAMYCQLIFSNTSVPSAIVSKCVAFFWNAAKKQHS

KSSVHAELRELPECTANLIENSHADDVFSPNMVERLWVLAKCTRDSAQMSDSIISSLSDVLVLSPLEVLA

SWYAADLLDALLMESLSRKVEISEIEEIISLCPKNSSIIRHALLAKLVLFPENTADSLNEVLAAYKNTLD

LCSQDKRKQSSVLKINLSKLFTLHSCLSLALQRLGYGDVSKRMYQEIFVPDSDADITPLSFIISWTALNT

FAPICTSPKENDVVEKMAMYVRTAIGTLKIQDLKLSRKLINSCIDIGSRLQEDLGYVSSA

>*Cneoformans* ([XP_567526)](http://www.ncbi.nlm.nih.gov/protein/58260232?report=genbank&log$=prottop&blast_rank=1&RID=7YHC7RKF013)

MPTSLYNTQFDQEEQDLIFAPSDVSPTDALFPPELTALFSDNQFPLDNPSNNFGGNHQHRGSLSSNEDSL

PFLSPTNPQSMSSTSPRLSLSPSTGEPSFHLTSHYSYDQSSHSPHSHSSASPLSSSGTDDVFLARSFEGV

SGGELDMFLLSEPINDYPVNSDGFGGQKPGQSLFLPGRTEGFNGMGDEWIQDLFKDPLQENGLDNINQQP

QPQPDGLDFDPILAGLQQRPQQQPLQQQKQQFGQTIQPQMVKQEPLIKQDSWSNGQNLSNSQHGFTFTHP

PFAPRNQAQDIKPASIPRTASVRRPTASKSETITAAAPAPTIGKHNKTERRYRQKVQAAQADLRDAIPAL

RLLYGTSTPEQLATTDIRAPDGTVDGLGEVTRPNASAKATILIGARVYIELLQKRSAKLQRMVNELEKFR

GAVEGEAGLAAWKEDFGRREAEIDRVEAEQLAAKLKEEEDESEGEDDDDDEEPSKKRKRTAAPKAKPKTA

AKTKITPQSTAAAGARVFAAFAMSFSFVPSASTVFRSNPQPQALATDKVLGYATNQQILASVPLIIAEHT

SRLLARSLPSALVPLPTTLLEWTWRLVVAVVLAVVMRPIISKLTTKSDEKARPGTVKGVLKDVVGIVSRK

KAQKTEWERFAAGVVGRANNVSTLAKWHTILHLNVTASSPYSLALLALLQPECSLLRSPQQLWLTAQSRV

DGTTPPALVTVLGLPLHEALLCLSSLPPTSAPLSALAEQITLIHVHDLYTRLFIHLVEASTASPLSTTSL

KSLLSALESHNLGANLKASAFDKEIKSVMQGTQKGSVVHALGLVLIGLWGIFTGPSSSAQASLAGALAAD

QVSGATHGLNSVSALLELLYPGCAPVSQAPAFVNPLSPNAQKVDALALSIIEYLSLLLTASRSTEATNMD

REGRKEESLGVQRKVVKLRGVLNKTGWVGVESQLDADDFEEVYDQPEEFTLGDEADKRDFRPHQLESERI

HYERAKERLVDILARIGRRAAGRANGRDEDSGLEGDLDEL

>*Hsapiens* ([P36956)](http://www.ncbi.nlm.nih.gov/protein/58260232?report=genbank&log$=prottop&blast_rank=1&RID=7YHC7RKF013)

MDEPPFSEAALEQALGEPCDLDAALLTDIEDMLQLINNQDSDFPGLFDPPYAGSGAGGTDPASPDTSSPG

SLSPPPATLSSSLEAFLSGPQAAPSPLSPPQPAPTPLKMYPSMPAFSPGPGIKEESVPLSILQTPTPQPL

PGALLPQSFPAPAPPQFSSTPVLGYPSPPGGFSTGSPPGNTQQPLPGLPLASPPGVPPVSLHTQVQSVVP

QQLLTVTAAPTAAPVTTTVTSQIQQVPVLLQPHFIKADSLLLTAMKTDGATVKAAGLSPLVSGTTVQTGP

LPTLVSGGTILATVPLVVDAEKLPINRLAAGSKAPASAQSRGEKRTAHNAIEKRYRSSINDKIIELKDLV

VGTEAKLNKSAVLRKAIDYIRFLQHSNQKLKQENLSLRTAVHKSKSLKDLVSACGSGGNTDVLMEGVKTE

VEDTLTPPPSDAGSPFQSSPLSLGSRGSGSGGSGSDSEPDSPVFEDSKAKPEQRPSLHSRGMLDRSRLAL

CTLVFLCLSCNPLASLLGARGLPSPSDTTSVYHSPGRNVLGTESRDGPGWAQWLLPPVVWLLNGLLVLVS

LVLLFVYGEPVTRPHSGPAVYFWRHRKQADLDLARGDFAQAAQQLWLALRALGRPLPTSHLDLACSLLWN

LIRHLLQRLWVGRWLAGRAGGLQQDCALRVDASASARDAALVYHKLHQLHTMGKHTGGHLTATNLALSAL

NLAECAGDAVSVATLAEIYVAAALRVKTSLPRALHFLTRFFLSSARQACLAQSGSVPPAMQWLCHPVGHR

FFVDGDWSVLSTPWESLYSLAGNPVDPLAQVTQLFREHLLERALNCVTQPNPSPGSADGDKEFSDALGYL

QLLNSCSDAAGAPAYSFSISSSMATTTGVDPVAKWWASLTAVVIHWLRRDEEAAERLCPLVEHLPRVLQE

SERPLPRAALHSFKAARALLGCAKAESGPASLTICEKASGYLQDSLATTPASSSIDKAVQLFLCDLLLVV

RTSLWRQQQPPAPAPAAQGTSSRPQASALELRGFQRDLSSLRRLAQSFRPAMRRVFLHEATARLMAGASP

TRTHQLLDRSLRRRAGPGGKGGAVAELEPRPTRREHAEALLLASCYLPPGFLSAPGQRVGMLAEAARTLE

KLGDRRLLHDCQQMLMRLGGGTTVTSS

**CLUSTAL X (1.83) multiple sequence alignment**

*Pb*18 ------------------------------------------------------------

*Pb*03 MRRKPPATKDRARSNEIKIIDCQTAAATITQPAHYPAQTPSTTPSTTPSKYTASTQQVPP 60

*Pb*01 ------------------------------------------------------------

*Afumigatus* ------------------------------------------------------------

*Spombe* ------------------------------------------------------------

*Hsapiens* ------------------------------------------------------------

*Cneoformans* ------------------------------------------------------------

*Pb*18 ------------------------------------------------------------

*Pb*03 ASTPSTLPTQHLVPSAATPPYPIPSTPRTQYPATPRTHQRPTRNPPNRPPPNRPCPRPRT 120

*Pb*01 ------------------------------------------------------------

*Afumigatus* ------------------------------------------------------------

*Spombe* ------------------------------------------------------------

*Hsapiens* ------------------------------------------------------------

*Cneoformans* ------------------------------------------------------------

*Pb*18 ------------------------------------------------------------

*Pb*03 GRPPAARDKELRRPTSKGERRRTKDQRRSLGGAGQSGPIFTPTASPPPLTRDEETDRNRS 180

*Pb*01 ------------------------------------------------------------

*Afumigatus* ------------------------------------------------------------

*Spombe* ------------------------------------------------------------

*Hsapiens* ------------------------------------------------------------

*Cneoformans* ------------------------------------------------------------

*Pb*18 --------------------------MSSSQSPLRVSMSPSTDFSSNNSPPQALTSDWSQ 34

*Pb*03 FIVRLYALISKREPAFYLLICLFVIAMSSSQSPLRVSMSPSTDFSSNNSPPQALTSDWSQ 240

*Pb*01 --------------------------MSFSQSPLRVSMSPSTDISSNNSPRQALTSDWSQ 34

*Afumigatus* --------------MSTPGIGGDFQLFSPLESTRRISQGNS--LSVDQSSTDVASQDWTQ 44

*Spombe* --------------------------MQSSIPSVSVSVASPAMETPTKASPDSKSPNSVG 34

*Hsapiens* ---------------MDEPPFSEAALEQALGEPCDLDAALLTDIEDMLQLINNQDSDFPG 45

*Cneoformans* ------------------------MPTSLYNTQFDQEEQDLIFAPSDVSPTDALFPPELT 36

. . :

*Pb*18 WMH-----------------LPPKTEIKTIDTNHTLPLKCFDPPPPVDSAFTFTAGSLPL 77

*Pb*03 WMQWDGFESDDISGHTDLFSLPPKTEIKTIDTNHTLPLKCFDPPPPVDSAFTFTAGSLPL 300

*Pb*01 WMQWDGFESDDISGHTDLFSLPPKTEIKTIDTNQTLPLKSFDPPPPVDSAFTFTAGSLPI 94

*Afumigatus* WMRWD-------------------DEQAFPETANASPSSPFDLAFISPSASSGREASDAM 85

*Spombe* AIPSS------------------SPLASSTKASTSTPFVENCSNLLCDLASIVEDSPPTL 76

*Hsapiens* LFDPPYAGSGAGGTDPASPDTSSPGSLSPPPATLSSSLEAFLSGPQAAPSPLSPPQPAPT 105

*Cneoformans* ALFSD-------------------NQFPLDNPSNNFGGNHQHRGSLSSNEDSLPFLSPTN 77

: . . .

*Pb*18 CETVSPQEPSCVSDTSPAPSDFFDSALSPGVADGRWNNSGATIPLPADNHRTK-PPEGKK 136

*Pb*03 CETVSPQEPSCVSDTSPAPSDFFDSALSPGVADGRWNNSGATIPLPADNHRTK-PPEGKK 359

*Pb*01 CETISPQEPSCVSDTSPAPSDFFDSALSPGVADGRWNKSGATIPLPPDNNRSKQPLEGKK 154

*Afumigatus* HKDFSPDIS--LDFKSPSLGFFPGGDLNTNVSP------------QPDHVGAG------S 125

*Spombe* TNTSLSPHSFSLSDMESSMSNWLNPFAFDNTMNSAPPLFTSTNMGSPNSLENSTNPLLSN 136

*Hsapiens* PLKMYPSMPAFSPGPGIKEESVPLSILQTPTPQPLPGALLPQSFPAPAPPQFSSTPVLGY 165

*Cneoformans* PQSMSSTSPRLSLSPSTGEPSFHLTSHYSYDQS------------SHSPHSHSSASPLSS 125

. .

*Pb*18 CSLHPSAPLPSFPITDRKRKTRPDEPPGSAGAGDSPVTSSKNLPSKKRSHNVIEKRYRAN 196

*Pb*03 CSLHPSAPLPSFPITDRKRKTRPDEPPGSAGAGYSPVTSSKNLPSKKRSHNVIEKRYRAN 419

*Pb*01 CSL-PSAPLPSFPITDRKRKTRPDEPPGSAGADDSPVSSSKNLPSKKRSHNVIEKRYRAN 213

*Afumigatus* LSVHSNSPLSSIGAS-RKRKTGSDD----DGSTMTSMFKAKQAPSKKRAHNVIEKRYRAN 180

*Spombe* CGSPNSFQNETFTGPSLNEFDADDKIQRQMKILHSVDTIDPSTVQNYPSADMSNPEVKLK 196

*Hsapiens* PSPPGGFSTGSPPGNTQQPLPGLPLASPPGVPPVSLHTQVQSVVPQQLLTVTAAPTAAPV 225

*Cneoformans* SGTDDVFLARSFEGVSGGELDMFLLSEPINDYPVNSDGFGGQKPGQSLFLPGRTEGFNGM 185

. : . . :

*Pb*18 LNEKIAELRDSVPSLRALAKQKSGISCQAVQDDDEIVSSSNKLNKASILSKATEYIRHLE 256

*Pb*03 LNEKIAELRDSVPSLRALAKQKSGISCQAVQDDDEIVSSSNKLNKASILSKATEYIRHLE 479

*Pb*01 LNEKIAELRDSVPSLRALAKQKSGISCQAVQDDDEIVSSSNKLNKASILSKATEYIRHLE 273

*Afumigatus* LNEKIAELRDSVPSLRASYKQANGNSG---DDDDDGVTSASKLNKASILSKATEYIRHLE 237

*Spombe* TEEIITPMDTTCKPEPSAKKIKLSPSSEDSCSIPETLPFSAPKSRGSLSPSETPDFVAGP 256

*Hsapiens* TTTVTSQIQQVPVLLQPHFIKADSLLLTAMKTDGATVKAAGLSPLVSGTTVQTGPLPTLV 285

*Cneoformans* GDEWIQDLFKDPLQENGLDNINQQPQPQPDGLDFDPILAGLQQRPQQQPLQQQKQQFGQT 245

: : . .

*Pb*18 LRNKRLEEENVALKNRLRQLEKVQEQSVVNYASMSGSVSSPDAYTVSTDSGTGSSPGVFS 316

*Pb*03 LRNKRLEEENVALKNRLRQLEKVQEQSVVNYASMSGSVSSPDAYTVSTDSGTGSSPGVFS 539

*Pb*01 LRNKRLEEENVALKNRLRQLEKVQEQNVVNYASTSGSVSSPDAYTVSTDSGAGSSPGVFS 333

*Afumigatus* IRNKRLEEENTALKIRLRQLDKAADQIVT----SAASVSSPSDCTVSTESGASSSPSVFS 293

*Spombe* GGKPKKTAHNMIEKRYRTNLNDRICELRDAVPSLRAAAALRCGNSLDDEDLGGLTP---A 313

*Hsapiens* SGGTILATVPLVVDAEKLPINRLAAGSKAPASAQSRGEKRTAHNAIEKRYRSSINDKIIE 345

*Cneoformans* IQPQMVKQEPLIKQDSWSNGQNLSNSQHGFTFTHPPFAPRNQAQDIKPASIPRTASVRRP 305

. : :.

*Pb*18 QNDDPS-PESSPNPLYPPEGLIKVPEYFKRMRMTGP-QPHYADSFIQRPNTTTTTYTSSE 374

*Pb*03 QNDDPS-PESSPNPLYPPEGLIKVPEYFKRMRMTGP-QPHYADSFIQRPNTTTTTYTSSE 597

*Pb*01 QNEDTS-PESSPNPLYPPEGLIKVPEYFKRMRMTGP-QPHYADSFIQRPNAN-TTYTSSE 390

*Afumigatus* HAEDVP-SDHSPTSSHPPEGLIKVPDAWKRMRAAGSNESPYSQSYIQYKKTDSHSSQSGG 352

*Spombe* RKLNKG-TILAKATEYIRHLEAKNKELQKTNKQLSDRLAFYEDPSMAPPSNDTRAVNSVN 372

*Hsapiens* LKDLVVGTEAKLNKSAVLRKAIDYIRFLQHSNQKLKQENLSLRTAVHKSKSLKDLVSACG 405

*Cneoformans* TASKSE-TITAAAPAPTIGKHNKTERRYRQKVQAAQADLRDAIPALRLLYGTSTPEQLAT 364

. . : . :

*Pb*18 -RKRTFIPNKFMLGTLAALMIVGGFESHK-SESSEKGLLGLPLQFAGEIHRFCQTHLCFP 432

*Pb*03 -RKRTFIPNKFMLGTLAALMIVGGFESHK-SESSEKGLLGLPLQFAGEIHRFCQTHLCFP 655

*Pb*01 -RKGTIIPNKFMLGTLAALMIVGGFESQK-SESSEKGLLGIPLQFAGEIHRFCQTHLCFP 448

*Afumigatus* GRMRSHLPNKYMLGALAGLMVLEGLGTEKKTESTAKGLLAVPLNLLNRVQLPSEVYSSAA 412

*Spombe* VVSSSDYSVHQSSRPNLTQRAFTSPTLNTMGRTALNGMVGLGLFNYFGNDSSQSVYGLFA 432

*Hsapiens* SGGNTDVLMEGVKTEVEDTLTPPPSDAGSPFQSSPLSLGSRGSGSGGSGSDSEPDSPVFE 465

*Cneoformans* TDIRAPDGTVDGLGEVTRPNASAKATILIGARVYIELLQKRSAKLQRMVNELEKFRGAVE 424

: . :

*Pb*18 TT---TSWQIRALSHFALTSLVVVGAAFAVFLYLFNS--APRRA--KHPAKSTTGSRNPA 485

*Pb*03 TT---TSWQIRALSHFALTSLVVVGAAFAVFLYLFNS--APRRA--KHPAKSTTGSRNPA 708

*Pb*01 TT---TSWQIRALSHFALTSLVVVGAAFAVFLYLFNS--APRRA--KHPAKSTTGSRNPA 501

*Afumigatus* FQYFWSSWHARAISHFLMLAILVVGSAFIVFVYLFNS--DPRRQ--YSASKVAP----DV 464

*Spombe* LP----PFLMSPFTGTVLFNMLKIGVVLLGLFYLLHD--NSLFKGFKGEKKSKVSTRSSM 486

*Hsapiens* DSK-AKPEQRPSLHSRGMLDRSRLALCTLVFLCLSCNPLASLLGARGLPSPSDTTSVYHS 524

*Cneoformans* GEAGLAAWKEDFGRREAEIDRVEAEQLAAKLKEEEDES-EGEDDDDDEEPSKKRKRTAAP 483

. : .

*Pb*18 AVSPIEFRREAWLT-SIQTVWVPSHNFFPEWFAVTWRCMEYVLSCILGWKLYSWLTGITE 544

*Pb*03 AVSPIEFRREAWLT-SIQTVWVPSHNFFPEWFAVTWRCMEYVLSCILGWKLYSWLTGITE 767

*Pb*01 AVSPIEFRREAWLT-SIQTVWVPSHNFFPEWFAVTWRCMEYVLSCILGWKLYSWLTGITE 560

*Afumigatus* TLSSCNFRRQAWLT-SIQRVGVPRHRFFHEWYVVTSRCFEYVLRCLLGWKLYSLVTGVTE 523

*Spombe* SPSSILFRKTVFEKYCLLDHSTSTISLFFGLLIFTLKSAYGYLTHRLSALYTSSENWVYS 546

*Hsapiens* PGRNVLGTESRDGPGWAQWLLPPVVWLLNGLLVLVSLVLLFVYGEPVTRPHSGPAVYFWR 584

*Cneoformans* KAKPKTAAKTKITPQSTAAAGARVFAAFAMSFSFVPSASTVFRSNPQPQALATDKVLGYA 543

. : ..

*Pb*18 EDETARAKAWD---IAIDAQLTGGDPEVSKSRMVLTIFAAGTLPSTPARVMLKALHCR-- 599

*Pb*03 EDETARAKAWD---IAIDAQLTGGDPEVSKSRMVLTIFAAGTLPSTPARVMLKALHCR-- 822

*Pb*01 EDETARAKAWD---IAIDAQLTGGDPEVSKSRMVLTIFAAGTLPSTPARVMLKALHCR-- 615

*Afumigatus* EDEKGRVKTWD---IAIDAQLAGGDAEISKSRLVLTIFAAGTLPRSPMRMMLKALHCR-- 578

*Spombe* EQQLAEVRNME---KLLDAQLMGGDAKVDRLRLLMVFASSFSLPPSSHTCALQAMYCQ-- 601

*Hsapiens* HRKQADLDLARGDFAQAAQQLWLALRALGRPLPTSHLDLACSLLWNLIRHLLQRLWVGRW 644

*Cneoformans* TNQQILASVPLIIAEHTSRLLARSLPSALVPLPTTLLEWTWRLVVAVVLAVVMRPIIS-- 601

: * . : : * :

*Pb*18 -----------LLLWMVGSRGSWAFRLSNHIAFLLASYQWEIARNLQKSLPKDHDDSLPD 648

*Pb*03 -----------LLLWMVGSRGSWAFRLSNHIAFLLASYQWEIARNLQKSLPKDHDDSLPD 871

*Pb*01 -----------LLLWMVGSRGSWAFRLSNHIAFLLASYQWEIARNLQKSLPKDHDDSLPD 664

*Afumigatus* -----------ILMWRVGEPGSWTFNVSNDVARSLARYQWDLARKMNAALPKDHPDSLPS 627

*Spombe* -----------LIFSNTSVP-------SAIVSKCVAFFWNAAKKQHSKSSVHAELRELPE 643

*Hsapiens* LAGRAGGLQQDCALRVDASASARDAALVYHKLHQLHTMGKHTGGHLTATNLALSALNLAE 704

*Cneoformans* ------------KLTTKSDEKARPGTVKGVLKDVVGIVSRKKAQKTEWERFAAGVVGRAN 649

: . : : ..

*Pb*18 HLATLLD-ANIDDVMTDAIIQRASNMAWNRCTQEATYGEDAMLDIVVEDTAVRSPLDALA 707

*Pb*03 HLATLLD-ANIDDVMTDAIIQRASNMAWNRCTQEATYGEDAMLDIVVEDTAVRSPLDALA 930

*Pb*01 HLATLLD-ANIDDVMTDAIIQRASNMAWNRCTQEATYGEDAMLDIVVEDTAVRSPLDALA 723

*Afumigatus* HLATLLK-IDCDDVMIDTIIQRAANLTWNRPTQEGTDDDEALLDVVEEDPAIQSSLDALA 686

*Spombe* CTANLIENSHADDVFSPNMVERLWVLAK--CTRDSAQMSDSIISSLS-DVLVLSPLEVLA 700

*Hsapiens* CAGDAVSVATLAEIYVAAALRVKTSLPRALHFLTRFFLSSARQACLAQSGSVPPAMQWLC 764

*Cneoformans* NVSTLAKWHTILHLNVTASSPYSLALLALLQPECSLLRSPQQLWLTAQSRVDGTTPPALV 709

. . .: : . . .. *

*Pb*18 AWWSSRALQN----------ALIHCLDVDTVEPSAQRTK-AFEQGLTLALNVAPIA---- 752

*Pb*03 AWWSSRALQN----------ALIHCLDVDTVEPSAQRTK-AFEQGLTLALNVAPIA---- 975

*Pb*01 AWWSSRALQN----------ALIHCLDVDTAEPSAQRTK-AFEQGLTLALNVAPIA---- 768

*Afumigatus* AWWSSHLLQG----------ALLRYFEASSGGPDAKKSRNVFKSKIKLALDVAPQP---- 732

*Spombe* SWYAADLLDA----------LLMESLSRKVEIS-----------EIEEIISLCPKN---- 735

*Hsapiens* HPVGHRFFVDGDWSVLSTPWESLYSLAGNPVDPLAQVTQLFREHLLERALNCVTQPNPSP 824

*Cneoformans* TVLGLPLHEA------------LLCLSSLPPTSAP-------LSALAEQITLIHVH---- 746

. : : . : :

*Pb*18 ---------SAAYTRAAVIKAVFFDEDRVANINTTLAALPRSKSQPAIS-STNFLDSSVP 802

*Pb*03 ---------SAAYTRAAVIKAVFFDEDRVANINTTLAALPRSKSQPAIS-STNFLDSSVP 1025

*Pb*01 ---------SAAYTRAAVIKAVFFDEDRVANINTTLAALPRSKSQSAIS-STNFLDSSVP 818

*Afumigatus* ---------SAAHTRALVMMAVFFERDRVANIGSVLAALPKEKGKNKQNQASNFLDSSLP 783

*Spombe* ---------SSIIRHALLAKLVLFPENTADSLNEVLAAYKNTLDLCSQD---KRKQSSVL 783

*Hsapiens* GSADGDKEFSDALGYLQLLNSCSDAAGAPAYSFSISSSMATTTGVDPVAKWWASLTAVVI 884

*Cneoformans* ------------DLYTRLFIHLVEASTASPLSTTSLKSLLSALESHNLG---ANLKASAF 791

: : :

*Pb*18 PSARAEISIAVRCAMIAAILRGQVDTNPVTSPQLTLENAITLFNILPVDPVELTLLGFAS 862

*Pb*03 PSARAEISIAVRCAMIAAILRGQVDTNPVTSPQLTLENAITLFNILPVDPVELTLLGFAS 1085

*Pb*01 PSARAEISIAVRCAMIAAILRGQVDTNPVTSPQLTLENAITLFNILPVDPVELTLLGFAS 878

*Afumigatus* ISVREEISTAVRCAMIAAIFNARATGDTSLPATFTVEKAIHWFNRLPLDPVELTLLEFAA 843

*Spombe* KINLSKLFTLHSCLSLALQRLGYG----------DVSKRMYQEIFVPDSDADITPLSFII 833

*Hsapiens* HWLRRDEEAAERLCPLVEHLPRVLQESERPLPRAALHSFKAARALLGCAKAESGPASLTI 944

*Cneoformans* DKEIKSVMQGTQKGSVVHALGLVLIGLWGIFTGPSSSAQASLAGALAADQVSGATHGLNS 851

. :. : .. :

*Pb*18 LYHLLHVVAAEPWMVLG-----SSLSSPISSSSSDTSSARQDGESSDTDGH----TLPTP 913

*Pb*03 LYHLLHVVAAEPWMVLG-----SSLSSPISSSSSDTSSARQDGESSDTDGH----TLPTP 1136

*Pb*01 LYHLLHIVAAEPWMVLG-----SSLSSPISSSSSDTSSARQDGESSDIDCH----PLPTP 929

*Afumigatus* VYHLLHILASDIDYLASSD--SSAPPSPMSKASDMLSSSSDDGEDGASQRNNNIIPHPMP 901

*Spombe* SWTALNTFA------------------PICTS---------------------------P 848

*Hsapiens* CEKASGYLQDSLATTPASSSIDKAVQLFLCDLLLVVRTSLWRQQQPPAPAPAAQGTSSRP 1004

*Cneoformans* VSALLELLYPG--------------CAPVSQAPAFVNPLSPNAQKVDALALSIIEYLSLL 897

. :.

*Pb*18 DLARIASGLIYWVRNAYNPVSSGFDMELVEKAVKGCVDVCQSVGIPIEAAEIQWEGHLVN 973

*Pb*03 DLARIASGLIYWVRNAYNPVSSGFDMELVEKAVKGCVDVCQSVGIPIEAAEIQWEGHLVN 1196

*Pb*01 DLARIASGLIYWVRNAYNPVSSGFDMELAEKAVKGCVDVCQSVGIHIEAAETKWEGHLVD 989

*Afumigatus* NLGRVASELIYWARNAYNPAFYGFTSNLVKVIETECTSLCQTAGVHVADYSCVQE----- 956

*Spombe* KENDVVEKMAMYVRTAIG-TLKIQDLKLSRKLINSCIDIGSRLQEDLGYVSSA------- 900

*Hsapiens* QASALELRGFQRDLSSLRRLAQSFRPAMRRVFLHEATARLMAGASPTRTHQLLDRSLRRR 1064

*Cneoformans* LTASRSTEATNMDREGRKEESLGVQRKVVKLRGVLNKTGWVGVESQLDADDFEEVYDQPE 957

. : . .

*Pb*18 NSAQGSQITQSTASEGRTSHSGDGGWEESQQNVENVRRESLESTDTGYGSLSQEDERADN 1033

*Pb*03 NSAQGSQITQSTASEGRTSHSGDGGWEESQQNVENVRRESLESTDTGYGSLSQEDERADN 1256

*Pb*01 NSAQGSQITQSTASEGRTNHSGDGGWEESQQNVENVRRESLESTDTGYGSLSQEDERADN 1049

*Afumigatus* ---EKSKAKQAIDSKRR----FAGGNEEASDNLL----------------LSDES----- 988

*Spombe* ------------------------------------------------------------

*Hsapiens* AGPGGKGGAVAELEPRPTRREHAEALLLASCYLPPGFLSAPGQRVGMLAEAARTLEKLGD 1124

*Cneoformans* EFTLGDEADKRDFRPHQLESERIHYERAKERLVDILARIGRRAAGRANGRDEDSGLEGDL 1017

*Pb*18 KEIKITATQRVEGVLSHQLGSSR 1056

*Pb*03 KEIKITATQRVEGVLSHQLGSSG 1279

*Pb*01 KEKKITATQRVEGVYLTN----- 1067

*Afumigatus* -----------------------

*Spombe* -----------------------

*Hsapiens* RRLLHDCQQMLMRLGGGTTVTSS 1147

*Cneoformans* DEL-------------------- 1020

- bHLH domain and protein length of proteins were determined using **SMART (****http://smart.embl-heidelberg.de):**

aa – amino acid

**bHLH domain:**

*Pb*01 – 204 to 278aa

*Pb*03 – 410 to 484aa

*Pb*18 – 187 to 261aa

*Af* – 171 to 242aa

*Sp* – 266 to 338aa

*Cn* – 326 to 408aa

*Hs* – 329 to 379aa

**Protein length:**

*Pb*01 – 1067aa

*Pb*03 – 1279aa

*Pb*18 – 1056aa

*Af* – 988aa

*Sp* – 900aa

*Cn* – 1020aa

*Hs* – 1147aa

- Transmembrane predicted domains using both, **Phobius (****<http://phobius.sbc.su.se/>)**and **SACS MEMSAT2 Prediction (****<http://www.sacs.ucsf.edu/cgi-bin/memsat.py>):**

*Pb*01 – (Phobius)457 to 480aa

(SACS MEMSAT2)462 to 480aa

*Pb*03 - (Phobius)602 to 621aa and 668 to 687aa

(SACS MEMSAT2)669 to 687aa

*Pb*18 – (Phobius)384 to 401aa and 441 to 464aa

(SACS MEMSAT2) 446 to 464aa

*Af* – (Phobius)428 to 477aa

(SACS MEMSAT2) 424 to 447aa

*Sp* – (Phobius)445 to 463aa and 510 to 529aa

(SACS MEMSAT2)441 to 463aa and 501 to 522aa

*Cn* –(Phobius)582 to 600aa and 805 to 822aa

(SACS MEMSAT2)805 to 822aa

*Hs* (SREBP2)–(Phobius)488 to 509aa and 548 to 567aa

(SACS MEMSAT2)488 to 509aa and 543 to 567aa
