# Supplementary material for: Characterization of the Paracoccidioides Hypoxia Response Reveals New Insights into Pathogenesis Mechanisms of This Important Human Pathogenic Fungus
Source: PLoS Negl Trop Dis. 2015 Dec 10;9(12):e0004282. doi: 10.1371/journal.pntd.0004282 (PMC4686304; doi:10.1371/journal.pntd.0004282)
Supplement: S1 Table — (DOCX) [file pntd.0004282.s005.docx]

**S1 Table. Up-regulated proteins of *Paracoccidioides* (*Pb*01) yeast cells under oxygen deprivation for 12 and 24 h, detected by NanoUPLC_MS^E^ analysis.**

| Incubation time under hypoxia | ID^a^ | Annotation^b^ | Score AVG^c^ | Peptides  AVG^c^ | Fold change^d^ | Biological process^e^ |  |
| --- | --- | --- | --- | --- | --- | --- | --- |
| METABOLISM | | | | | | |  |
| Amino acid metabolism | | | | | | |  |
| 12 h | PAAG_00221 | acetolactate synthase | 569,68 | 6 | # | isoleucine biosynthesis |  |
| 12 h | PAAG_02170 | adenylosuccinate synthetase | 547,29 | 5 | # | aspartate biosynthesis |  |
| 12 h | PAAG_06387 | homoisocitrate dehydrogenase | 707,74 | 6 | # | isoleucine biosynthesis |  |
| 12 h | PAAG_02693 | saccharopine dehydrogenase | 535,06 | 9 | # | lysine biosynthesis |  |
| 12 h | PAAG_08701 | D-3-phosphoglycerate dehydrogenase | 492 | 4 | # | serine biosynthesis |  |
| 12 h | PAAG_07689 | NADP-specific glutamate dehydrogenase | 485,81 | 13 | 0.61 | glutamine degradation |  |
| 12 h | PAAG_00966 | L-threonine 3-dehydrogenase | 738,5 | 8 | # | threonine degradation |  |
| 24 h | PAAG_05328 | 3-isopropylmalate dehydrogenase A | 1003,73 | 6 | # | leucine biosynthesis |  |
| 24 h | PAAG_08166 | 4-hydroxyphenylpyruvate dioxygenase | 679,3 | 7 | # | glycine biosynthesis |  |
| 24 h | PAAG_07605 | acetolactate synthase small subunit | 953,28 | 7 | # | valin, leucine and isoleucine biosynthesis |  |
| 24 h | PAAG_09095 | ATP phosphoribosyltransferase | 497,15 | 5 | # | L-histidine biosynthesis |  |
| 24 h | PAAG_03537* | lysine decarboxylase-like protein | 811,54 | 4 | # | Lysine biosynthesis |  |
| 24 h | PAAG_08701 | D-3-phosphoglycerate dehydrogenase | 440,33 | 6 | # | L-serine biosynthesis |  |
| 24 h | PAAG_05984 | glutaryl-CoA dehydrogenase | 517,15 | 7 | # | L-tryptophan degradation |  |
| 24 h | PAAG_05406 | histidine biosynthesis trifunctional protein | 532,97 | 8 | # | histidine biosynthesis |  |
| 24 h | PAAG_06387 | homoisocitrate dehydrogenase | 458,29 | 8 | # | isoleucine biosynthesis |  |
| 24 h | PAAG_04348 | homoserine kinase | 771,49 | 4 | # | L-threonine degradation |  |
| 24 h | PAAG_02693 | saccharopine dehydrogenase | 463,32 | 9 | # | lysine biosynthetic process via aminoadipic acid |  |
| C-compound and carbohydrate metabolism | | | | | | |  |
| 12 h | PAAG_03910 | aldehyde dehydrogenase | 414,74 | 10 | # | acetyl-CoA from acetaldehyde |  |
| 24 h | PAAG_02938 | glucosamine 6-phosphate synthetase | 464,63 | 5 | # | carbohydrate biosynthetic process/ carbohydrate binding |  |
| 24 h | PAAG_00545 | glycogen phosphorylase | 521,52 | 16 | # | glycogen catabolic process |  |
| 24 h | PAAG_00889 | phosphomannomutase | 535,81 | 6 | # | [GDP-mannose](http://www.ebi.ac.uk/QuickGO/GTerm?id=GO:0009298) metabolism |  |
| 24 h | PAAG_02718 | mannose-1-phosphate guanyltransferase | 653,52 | 7 | # | mannose metabolism |  |
| Nitrogen metabolism | | | | | | |  |
| 12 h | PAAG_08277 | nitroreductase family protein | 451,7 | 3 | # | reduction of nitrogen-containing compound |  |
| 24 h | PAAG_04233 | 2-nitropropane dioxygenase | 638,34 | 3 | # | nitrogen, sulfur and selenium metabolism |  |
| 24 h | PAAG_04966 | hydrolase | 570,91 | 6 | # | nitrilase homologue (detoxification of nitrogen compound)/ carbon-nitrogen hydrolase domain |  |
| Lipid, fatty acid and isoprenoid metabolism | | | | | | |  |
| 12 h | PAAG_07432* | [long-chain specific acyl-CoA dehydrogenase](http://www.ncbi.nlm.nih.gov/blast/Blast.cgi#alnHdr_225678794) | 1005,1 | 9 | # | beta-oxidation |  |
| 24 h | PAAG_06329 | 3-hydroxybutyryl-CoA dehydrogenase | 1509,07 | 5 | # | butanoate metabolism (3-acetoacetyl-CoA production) |  |
| 24 h | PAAG_07432* | long-chain specific acyl-CoA dehydrogenase | 805,07 | 11 | # | peroxissomal fatty acid beta-oxidation |  |
| 24 h | PAAG_01525 | fatty acid synthase subunit alpha reductase | 479,14 | 35 | # | lipid, fatty acid and isoprenoid metabolism |  |
| 24 h | PAAG_01524 | fatty acid synthase subunit beta dehydratase | 668,21 | 25 | # | lipid, fatty acid and isoprenoid metabolism |  |
| Purine nucleotide/ nucleoside/ nucleobase metabolism | | | | | | |  |
| 12 h | PAAG_02333 | GMP synthase | 1178,41 | 6 | # | purin nucleotide/nucleoside/nucleobase metabolism |  |
| 12 h | PAAG_08696* | [RdgB/HAM1 family non-canonical purine NTP pyrophosphatase](http://www.ncbi.nlm.nih.gov/blast/Blast.cgi#alnHdr_225562252) | 717,97 | 7 | # | purin nucleotide/nucleoside/nucleobase metabolism |  |
| 24 h | PAAG_05019* | Hit family protein 1 | 1428,71 | 4 | # | nucleotide metabolic process |  |
| 24 h | PAAG_05803 | inosine-5- monophosphate dehydrogenase IMD2 | 647,56 | 8 | # | purine anabolism |  |
| 24 h | PAAG_02115 | ribose-phosphate pyrophosphokinase | 599,01 | 12 | # | nucleotide biosynthetic process/ fungal-type cell wall organization |  |
| Phosphate metabolism | | | | | | |  |
| 24 h | PAAG_05610 | phosphotransferase enzyme family domain-containing protein | 566,71 | 8 | # | phosphate metabolism |  |
| 24 h | PAAG_03267 | phosphotransferase enzyme family protein | 469,95 | 10 | # | phosphate metabolism |  |
| 24 h | PAAG_08275 | phosphotransferase enzyme family protein | 11,15 | 1 | # | phosphate metabolism |  |
| Secondary metabolism | | | | | | |  |
| 12 h | PAAG_08856 | nicotinate-nucleotide pyrophosphorylase | 556,75 | 7 | # | biosynthesis of vitamins, cofactors, and prosthetic groups |  |
| 12 h | PAAG_05780 | 2,5-diketo-D-gluconic acid reductase A | 447,15 | 5 | # | L-ascorbic acid biosynthetic process (vitamin C) |  |
| 24 h | PAAG_05780 | 2,5-diketo-D-gluconic acid reductase A | 769,56 | 6 | # | ascorbate biosynthesis |  |
| ENERGY | | | | | | |  |
| Glycolysis and gluconeogenesis | | | | | | |  |
| 24 h | PAAG_07410 | 2,3-bisphosphoglycerate independent phosphoglycerate mutase | 454,11 | 9 | # | glycolysis/ pyruvate formation |  |
| 24 h | PAAG_06526 | glucose-6-phosphate isomerase | 587,73 | 9 | # | glycolysis |  |
| Electron transport and membrane-associated energy conservation | | | | | | |  |
| 12 h | PAAG_01603 | cytochrome b2 | 431,84 | 6 | # | L-lactate cytochrome c oxidoreductase activity |  |
| 24 h | PAAG_04820 | ATPase alpha subunit | 2048,5 | 16 | 0.71 | electron transport and membrane-associated energy conservation |  |
| 24 h | PAAG_05576 | ATP synthase gamma chain | 1218,74 | 7 | # | aerobic respiration |  |
| 24 h | PAAG_08037 | ATP synthase subunit beta | 3240,46 | 20 | 0.63 | aerobic respiration |  |
| 24 h | PAAG_08019 | adenylate kinase cytosolic | 486,58 | 6 | # | ADP biosynthetic process |  |
| 24 h | PAAG_02265 | mitochondrial F1F0 ATP synthase subunit | 500,99 | 1 | # | aerobic respiration |  |
| GABA shunt | | | | | | |  |
| 24 h | PAAG_07689 | NADP specific glutamate dehydrogenase | 644,86 | 9 | # | glutamine degradation |  |
| 24 h | PAAG_08718 | succinate-semialdehyde dehydrogenase | 624,8 | 10 | # | γ-aminobutyric acid or GABA degradation |  |
| CELL CYCLE and DNA PROCESSING | | | | | | |  |
| 12 h | PAAG_12288* | UDP-N-acetylglucosamine pyrophosphorylase | 1192,15 | 1 | # | DNA binding |  |
| 12 h | PAAG_00126 | histone H4.2 | 2255,6 | 5 | # | DNA processing |  |
| 24 h | PAAG_00209 | dynactin | 9,94 | 1 | 1.11 | nuclear division |  |
| 24 h | PAAG_05543* | SET domain-containing protein 5 | 628,13 | 3 | # | histone methylation |  |
| 24 h | PAAG_12254* | telomere-binding alpha subunit central domain-containing protein | 391,54 | 7 | # | telomere maintenance |  |
| 24 h | PAAG_06574 | curved DNA-binding protein 42 kDa protein | 1206,41 | 10 | # | DNA binding |  |
| 24 h | PAAG_03188 | nuclear movement protein nudC | 769,56 | 4 | # | mitotic nuclear division |  |
| 24 h | PAAG_02186 | nuclear segregation protein Bfr1 | 432,94 | 16 | # | mitotic cell cycle and cell cycle control |  |
| 24 h | PAAG_01986 | nucleosome binding protein | 978,36 | 1 | # | DNA conformation modification (e.g. chromatin) |  |
| 24 h | PAAG_08443* | meiosis protein MEI2 | 388,19 | 7 | # | meiosis/ cell cycle |  |
| 24 h | PAAG_00438 | Sad1/UNC domain-containing protein | 396,19 | 7 | # | nuclear matrix anchoring at nuclear membrane |  |
| TRANSCRIPTION | | | | | | |  |
| 12 h | PAAG_04496 | nascent polypeptide-associated complex subunit beta | 1053,77 | 3 | # | transcriptional control |  |
| 12 h | PAAG_12589* | C6 finger domain-containing protein | 423,71 | 8 | # | transcription |  |
| 24 h | PAAG_07594 | ATP-dependent RNA helicase DOB1 | 507,28 | 12 | # | rRNA processing |  |
| 24 h | PAAG_01285* | splicing factor-like protein | 449,59 | 4 | # | Transcription |  |
| 24 h | PAAG_04982 | transcription initiation factor IIE subunit beta | 384,89 | 4 | # | general transcription activities |  |
| TRANSLATION | | | | | | |  |
| 12 h | PAAG_02837 | eukaryotic translation initiation factor 3 subunit H | 1033,72 | 8 | # | protein biosynthesis |  |
| 12 h | PAAG_06627 | 60S ribosomal protein L32 | 1003,9 | 1 | # | ribosome biogenesis |  |
| 12 h | PAAG_11057* | arginine-tRNA ligase | 471,77 | 9 | # | aminoacyl-tRNA-synthetases |  |
| 12 h | PAAG_07349 | glycyl-tRNA synthetase | 491,17 | 12 | # | aminoacyl-tRNA-synthetases |  |
| 12 h | PAAG_07170 | eukaryotic translation initiation factor 3 135 kDa subunit | 575,18 | 14 | # | translate |  |
| 12 h | PAAG_04572 | ribosomal protein L14 | 1818,28 | 3 | # | ribosomal proteins |  |
| 12 h | PAAG_05805 | 40S ribosomal protein S21 | 3259,76 | 3 | # | ribosomal proteins |  |
| 12 h | PAAG_06627 | 60S ribosomal protein L32 | 1003,9 | 1 | # | ribosomal proteins |  |
| 12 h | PAAG_04998 | 60S ribosomal protein L8-B | 1505,47 | 15 | 0.75 | ribosome biogenesis |  |
| 12 h | PAAG_12263* | PCI domain-containing protein | 521,54 | 3 | # | [protein biosynthesis](http://www.ebi.ac.uk/QuickGO/GTerm?id=GO:0006368) |  |
| 12 h | PAAG_00815 | eukaryotic translation initiation factor 3 subunit A | 412,02 | 19 | # | translation initiation |  |
| 12 h | PAAG_02837 | eukaryotic translation initiation factor 3 subunit H | 1033,72 | 8 | # | translation initiation |  |
| 12 h | PAAG_05832 | eukaryotic translation initiation factor 2 ubunit alpha | 595,26 | 4 | # | translation initiation |  |
| 12 h | PAAG_06623 | translation initiation factor 4B | 519,03 | 7 | # | translation initiation |  |
| 12 h | PAAG_08348 | eukaryotic translation initiation factor 3 39 kDa subunit | 675,14 | 5 | # | translation initiation |  |
| 24 h | PAAG_05337 | 40S ribosomal protein S22 | 1630,64 | 6 | # | ribosomal proteins |  |
| 24 h | PAAG_00385 | 40S ribosomal protein S23 | 837,09 | 3 | # | ribosome biogenesis |  |
| 24 h | PAAG_08540 | 40S ribosomal protein S25 | 2288,73 | 3 | # | ribosomal proteins |  |
| 24 h | PAAG_05233 | 60S ribosomal protein L26 | 1589,52 | 4 | # | ribosome biogenesis |  |
| 24 h | PAAG_04201 | 60S ribosomal protein L27 | 649,48 | 4 | # | ribosome biogenesis |  |
| 24 h | PAAG_00088 | 60S ribosomal protein L3 | 1202,93 | 14 | # | ribosomal proteins |  |
| 24 h | PAAG_07550 | 60S ribosomal protein L44 | 983,09 | 3 | # | ribosomal proteins |  |
| 24 h | PAAG_04572 | ribosomal protein L14 | 2581,01 | 6 | # | ribosomal proteins |  |
| 24 h | PAAG_01777 | alanyl-tRNA synthetase | 405,75 | 16 | # | aminoacyl-tRNA-synthetases |  |
| 24 h | PAAG_04904 | ATP binding cassette sub-family F member 2 | 437,44 | 8 | # | positive regulation of translation |  |
| 24 h | PAAG_03167 | elongation factor G 1 | 652,13 | 12 | # | translation elongation |  |
| 24 h | PAAG_00338 | methionyl-tRNA synthetase | 510,92 | 12 | # | aminoacyl-tRNA-synthetases/ translation |  |
| 24 h | PAAG_01786 | phenylalanyl-tRNA synthetase beta chain | 633,3 | 10 | # | aminoacyl-tRNA-synthetases/ translation |  |
| 24 h | PAAG_03951 | prolyl-tRNA synthetase | 875,73 | 8 | # | aminoacyl-tRNA-synthetases/ translation |  |
| 24 h | PAAG_08702 | seryl-tRNA synthetase | 381,83 | 11 | # | aminoacyl-tRNA-synthetases/ translation |  |
| PROTEIN FATE | | | | | | |  |
| 12 h | PAAG_00739 | peptidyl-prolyl cis-trans isomerase B | 442,51 | 4 | 2.90 | protein folding and stabilization |  |
| 12 h | PAAG_11406* | 26S protease regulatory subunit 6B | 1326,4 | 4 | # | protein degradation |  |
| 12 h | PAAG_01104 | proteasome component PUP1 | 533,45 | 4 | # | proteasomal degradation (ubiquitin/proteasomal pathway) |  |
| 12 h | PAAG_03687 | proteasome component PUP2 | 432,88 | 6 | # | proteasomal degradation (ubiquitin/proteasomal pathway) |  |
| 12 h | PAAG_01727 | T-complex protein 1 subunit delta | 424,68 | 11 | # | protein folding and stabilization |  |
| 12 h | PAAG_06068 | T-complex protein 1 subunit beta | 461,78 | 13 | # | protein folding and stabilization |  |
| 12 h | PAAG_08020 | 26S proteasome regulatory subunit rpn-8 | 993,95 | 8 | # | protein/peptide degradation |  |
| 12 h | PAAG_11074* | ubiquitin-60S ribosomal protein L40 | 4146,04 | 5 | # | protein degradation |  |
| 12 h | PAAG_08205 | 26S proteasome non-ATPase regulatory subunit 6 | 413,32 | 10 | # | translation |  |
| 12 h | PAAG_01926* | [26S protease regulatory subunit 6A](http://www.ncbi.nlm.nih.gov/blast/Blast.cgi#alnHdr_226288798) | 650,31 | 4 | # | ubiquitin-dependent protein catabolic process |  |
| 24 h | PAAG_00770 | 26S protease regulatory subunit | 545,53 | 9 | # | proteasomal degradation (ubiquitin/proteasomal pathway) |  |
| 24 h | PAAG_02417 | ATP-dependent protease La 2 | 407,8 | 14 | # | protein/peptide degradation/ATP dependent |  |
| 24 h | PAAG_08205 | 26S proteasome non-ATPase regulatory subunit 6 | 466,52 | 10 | # | proteasome assembly |  |
| 24 h | PAAG_04674* | translocation protein Sec66 | 880,33 | 3 | # | protein targeting, sorting and translocation |  |
| 24 h | PAAG_05171 | peptidyl prolyl cis-trans isomerase | 394,48 | 7 | # | protein folding and stabilization |  |
| 24 h | PAAG_00866 | proteasome component C5 | 863,08 | 7 | # | proteasomal degradation (ubiquitin/proteasomal pathway) |  |
| 24 h | PAAG_01104 | proteasome component PUP1 | 496,21 | 5 | # | proteasomal degradation (ubiquitin/proteasomal pathway) |  |
| 24 h | PAAG_07851 | T-complex protein 1 subunit eta | 446,61 | 10 | # | protein folding and stabilization |  |
| 24 h | PAAG_08287 | T-complex protein 1 subunit theta | 525,81 | 9 | # | protein folding and stabilization |  |
| 24 h | PAAG_02773 | ubiquitin conjugating enzyme variant MMS2 | 1378,15 | 5 | # | protein polyubiquitination |  |
| 24 h | PAAG_07500 | xaa-Pro aminopeptidase | 689,54 | 10 | # | protein/peptide degradation |  |
| TRANSPORT | | | | | | |  |
| 12 h | PAAG_04605 | transport protein sec22 | 434,51 | 4 | # | vesicular transport (Golgi network, etc.) |  |
| 12 h | PAAG_01539* | [SNF7 family protein Fti1/Did2](http://www.ncbi.nlm.nih.gov/blast/Blast.cgi#alnHdr_239609140) | 1262,95 | 6 | # | protein transport |  |
| 12 h | PAAG_07175* | RAB GTPase Vps21/Ypt51 | 510,79 | 6 | # | intracelular protein transport |  |
| 12 h | PAAG_06878 | V-type ATPase, G subunit | 738,25 | 1 | # | Ion transport |  |
| 24 h | PAAG_05476 | ADP-ribosylation factor family protein | 946,6 | 4 | # | Protein transport/ ER to Golgi vesicle-mediated transport |  |
| 24 h | PAAG_03863 | acetyl-coenzyme A transporter 1 | 5,58 | 1 | # | vitamine/cofactor transport |  |
| 24 h | PAAG_00706* | gamma-taxilin | 685,43 | 9 | # | intracellular vesicle traffic (Nogami et al., 2003) |  |
| 24 h | PAAG_02625 | vesicle transport v-SNARE protein vti1 | 765,15 | 8 | # | protein targeting, sorting and translocation |  |
| 24 h | PAAG_03137 | vacuolar protein sorting-associated protein | 1079,8 | 3 | # | protein transport |  |
| CELL RESCUE, DEFENSE and VIRULENCE | | | | | | |  |
| 12 h | PAAG_02130 | heat shock protein HSP98 | 540,03 | 16 | # | stress response/ protein folding |  |
| 12 h | PAAG_06947 | gamma-glutamyltranspeptidase | 566,73 | 10 | # | disease, virulence and defense/ |  |
| 12 h | PAAG_01454 | catalase | 674,16 | 3 | # | oxidative stress response |  |
| 12 h | PAAG_05226 | Hsp90 binding co-chaperone (Sba1) | 396,52 | 2 | # | stress response/ protein folding and stabilization |  |
| 12 h | PAAG_07020 | thioredoxin reductase | 785,65 | 7 | # | stress response/ removal of superoxide radicals |  |
| 24 h | PAAG_03931 | glutathione S-transferase Gst3 | 670,42 | 4 | # | oxidation-reduction process/ removal of superoxide radicals |  |
| 24 h | PAAG_02116 | Hsp70 | 494,51 | 17 | # | stress response |  |
| 24 h | PAAG_08277 | nitroreductase family protein | 651,89 | 5 | # | response to toxic substance/ aromatic compound catabolic process |  |
| CELL GROWTH/ MORPHOGENESIS | | | | | | |  |
| 12 h | PAAG_05663 | ARP2 3 complex 34 kDa subunit | 579,94 | 8 | # | actin filament organization |  |
| 12 h | PAAG_07958 | fimbrin | 587,33 | 11 | # | budding, cell polarity and filament formation |  |
| 24 h | PAAG_02100 | glycolipid-anchored surface protein | 506,73 | 7 | # | cell wall organization |  |
| SIGNAL TRANSDUCTION | | | | | | |  |
| 12 h | PAAG_08247 | calmodulin | 843,77 | 4 | # | calcium binding |  |
| BINDING | | | | | | |  |
| 12 h | PAAG_09071* | RING finger domain-containing protein | 428,73 | 10 | # | RNA binding |  |
| 24 h | PAAG_05585* | Nuclease domain protein | 1321,39 | 7 | # | RNA binding |  |
| 24 h | PAAG_00963* | RING finger domain-containing protein | 578,24 | 8 | # | DNA, RNA, protein, zin ion and/or lipid binding |  |
| MISCELLANEOUS | | | | | | |  |
| 12 h | PAAG_06955* | [thiol methyltransferase](http://www.ncbi.nlm.nih.gov/blast/Blast.cgi#alnHdr_239606682) | 1243,85 | 8 | # | transferase |  |
| 24 h | PAAG_01870* | short-chain dehydrogenase reductase SDR | 602,84 | 5 | # | oxidoreductase |  |
| 24 h | PAAG_06083 | dienelactone hydrolase family protein | 422,35 | 4 | # | hydrolase activity |  |
| 24 h | PAAG_05134 | HAD superfamily hydrolase | 724,3 | 2 | # | hydrolase activity |  |
| 24 h | PAAG_06953 | short chain dehydrogenase/ reductase family | 3232,81 | 5 | # | oxidoreductase activity |  |
| UNCLASSIFIED | | | | | | | |
| 12 h | PAAG_00328 | IQ calmodulin-binding motif domain-containing protein | 18,39 | 1 | # | - |  |
| 12 h | PAAG_00870 | hypothetical protein | 610,73 | 3 | # | - |  |
| 12 h | PAAG_01045* | UPF0135 protein yqfO | 901,45 | 3 | # | - |  |
| 12 h | PAAG_01663 | hypothetical protein | 483,4 | 3 | # | - |  |
| 12 h | PAAG_02502 | conserved hypothetical protein | 411,32 | 11 | # | - |  |
| 12 h | PAAG_03239 | hypothetical protein | 421,29 | 3 | # | - |  |
| 12 h | PAAG_05281 | predicted protein | 1053,94 | 2 | # | - |  |
| 12 h | PAAG_05348 | DUF1014 domain-containing protein | 863,9 | 5 | # | - |  |
| 12 h | PAAG_07136 | hypothetical protein | 395,05 | 12 | # | - |  |
| 24 h | PAAG_03743 | conserved hypothetical protein | 950,39 | 2 | # | - |  |
| 24 h | PAAG_06622 | conserved hypothetical protein | 550,92 | 2 | # | - |  |
| 24 h | PAAG_04450 | conserved hypothetical protein | 479,11 | 1 | # | - |  |
| 24 h | PAAG_11743 | MFS alpha-glucoside transporter | 371,69 | 8 | # | - |  |
| 24 h | PAAG_04732 | conserved hypothetical protein | 481,31 | 6 | # | - |  |
| 24 h | PAAG_00335* | MYG1 protein | 589,5 | 6 | # | - |  |
| 24 h | PAAG_02105 | conserved hypothetical protein | 5,58 | 1 | # | - |  |
| 24 h | PAAG_03921 | HET-C domain-containing protein HetC | 45,39 | 1 | # | - |  |
| 24 h | PAAG_01591 | hypothetical protein | 2648,54 | 4 | # | - |  |
| 24 h | PAAG_02353 | predicted protein | 701,96 | 3 | # | - |  |
| 24 h | PAAG_02237 | predicted protein | 1093,7 | 2 | # | - |  |
| 24 h | PAAG_05819 | predicted protein | 402,84 | 1 | # | - |  |

^a^ Identification of differentially regulated proteins from *Paracoccidioides* genome database (<http://www.broadinstitute.org/annotation/genome/paracoccidioides_brasiliensis/MultiHome.html>) using the ProteinLynx Global Server (PLGS) version 3.0 (Waters Corporation. Manchester. UK);

^b^ Proteins annotation from *Paracoccidioides* genome database or by homology (indicated using “*”) from NCBI database (<http://www.ncbi.nlm.nih.gov/>);

^c^  Average of protein score and matched peptides for each protein obtained from MS data using the ProteinLynx Global Server (PLGS);

^d^ Protein expression profiles in log2 (fold change) obtained from ProteinLynx Global Server (PLGS) analysis normalized with internal standard.

^e^ Biological process of differentially expressed proteins from MIPS

(<http://pedant.helmholtz-muenchen.de/pedant3htmlview/pedant3view?Method=analysis&Db=p3_r48325_Par_brasi_Pb01> ) and Uniprot database (<http://www.uniprot.org/>).

#: identified only in hypoxia condition.
