# Supplementary material for: Characterization of the Paracoccidioides Hypoxia Response Reveals New Insights into Pathogenesis Mechanisms of This Important Human Pathogenic Fungus
Source: PLoS Negl Trop Dis. 2015 Dec 10;9(12):e0004282. doi: 10.1371/journal.pntd.0004282 (PMC4686304; doi:10.1371/journal.pntd.0004282)
Supplement: S2 Table — (DOCX) [file pntd.0004282.s006.docx]

**S2 Table. Down-regulated proteins of *Paracoccidioides* (*Pb*01) yeast cells under oxygen deprivation for 12 and 24 h, detected by NanoUPLC_MS^E^ analysis.**

| Incubation time under hypoxia | ID^a^ | Annotation^b^ | Score AVG^c^ | Peptides AVG^c^ | Fold change^d^ | Biological process^e^ |  |
| --- | --- | --- | --- | --- | --- | --- | --- |
| METABOLISM | | | | | | |  |
| Amino acid metabolism | | | | | | |  |
| 12 h | PAAG_02644 | kynurenine oxoglutarate transaminase | 806,25 | 9 | ** | glutamate biosynthesis |  |
| 12 h | PAAG_02603 | aspartate aminotransferase | 492,07 | 6 | -0.95 | amino acid metabolism |  |
| 12 h | PAAG_02115 | ribose phosphate pyrophosphokinase | 539,04 | 12 | ** | histidine and tryptophan biosynthesis |  |
| 12 h | PAAG_05253 | delta-1-pyrroline-5-carboxylate dehydrogenase | 1163,12 | 12 | -0.94 | glutamate biosynthesis |  |
| 12 h | PAAG_08649 | cysteine dioxygenase | 492,5 | 2 | ** | cysteine degradation |  |
| 12 h | PAAG_01310 | 2 -oxoisovalerate dehydrogenase subunit alpha, mitochondrial | 746,31 | 12 | ** | isoleucine degradation |  |
| 24 h | PAAG_05198 | chorismate mutase | 1185,6 | 6 | -0.62 | tyrosine and L-phenylalanine biosynthetic process |  |
| 24 h | PAAG_02603 | aspartate aminotransferase | 614,27 | 7 | ** | amino acid metabolism/ aspartate degradation process |  |
| 24 h | PAAG_01144 | aspartate aminotransferase | 1033,03 | 12 | ** | amino acid metabolism/ aspartate degradation process |  |
| 24 h | PAAG_05253 | delta-1-pyrroline-5-carboxylate dehydrogenase | 1343,48 | 11 | ** | glutamate biosynthesis |  |
| 24 h | PAAG_01310 | 2-oxoisovalerate dehydrogenase subunit alpha, mitochondrial | 729,7 | 12 | ** | degradation of isoleucine / conversion of alpha-keto acids to acyl-CoA and CO2 |  |
| 24 h | PAAG_03613 | phosphoserine aminotransferase | 759,59 | 9 | ** | L-serine biosynthesis |  |
| C-compound and carbohydrate metabolism | | | | | | |  |
| 12 h | PAAG_02769 | pyruvate dehydrogenase protein X component | 484 | 8 | ** | acetyl-CoA biosynthetic process from pyruvate |  |
| 12 h | PAAG_00545 | glycogen phosphorylase | 597,49 | 14 | -1.11 | glycogen catabolic process |  |
| 12 h | PAAG_00666* | pyruvate dehydrogenase complex component Pdx1 | 876,38 | 20 | ** | [acetyl-CoA biosynthetic process from pyruvate](http://www.ebi.ac.uk/QuickGO/GTerm?id=GO:0006086) |  |
| 12 h | PAAG_00726 | pyruvate carboxylase | 471,44 | 12 | ** | pyruvate metabolism/ oxaloacetate from pyruvate |  |
| 12 h | PAAG_00885 | acetylcholinesterase | 434,08 | 12 | ** | C-compound and carbohydrate metabolism |  |
| 12 h | PAAG_02718 | mannose-1-phosphate guanyltransferase | 445,16 | 8 | ** | mannose metabolism (phosphate + GDP-mannose production) |  |
| 12 h | PAAG_04761 | choline dehydrogenase | 443,52 | 6 | ** | C-compound and carbohydrate metabolism |  |
| 24 h | PAAG_11035 | pyruvate dehydrogenase protein X component | 768,31 | 9 | -0.68 | acetyl-CoA biosynthetic process from pyruvate |  |
| 24 h | PAAG_02653 | acetyl-coenzyme A synthetase | 536,54 | 4 | ** | acetyl-CoA biosynthetic process from acetate |  |
| 24 h | PAAG_00726 | pyruvate carboxylase | 526,47 | 21 | ** | pyruvate metabolism/ oxaloacetate from pyruvate |  |
| 24 h | PAAG_00666* | pyruvate dehydrogenase complex component Pdx1 | 607,59 | 4 | ** | C-compound and carbohydrate metabolism/ pyruvate metabolic process |  |
| 24 h | PAAG_05367 | alcohol dehydrogenase zinc-binding domain-containing protein | 1188,73 | 2 | ** | amino acid catabolic process to alcohol via Ehrlich pathway |  |
| 24 h | PAAG_08174 | mannose-1- phosphate guanyltransferase subunit beta-A | 790,86 | 7 | -0.72 | mannose metabolism (phosphate + GDP-mannose production) |  |
| 24 h | PAAG_12288* | UDP-N-acetylglucosamine pyrophosphorylase | 883,41 | 2 | ** | Nucleotide-sugar biosynthesis; UDP-N acetylglucosamine diphosphorylase activity |  |
| Nitrogen metabolism | | | | | | |  |
| 12 h | PAAG_05353 | flavoprotein oxygenase | 516,6 | 5 | ** | nitrogen, sulfur and selenium metabolism |  |
| 12 h | PAAG_07428 | allantoinase | 736,35 | 3 | ** | nitrogen, sulfur and selenium metabolism |  |
| Purine nucleotide/ nucleoside/ nucleobase metabolism | | | | | | |  |
| 12 h | PAAG_08420 | ribonuclease H2 subunit A | 466,68 | 8 | ** | polynucleotide degradation |  |
| 12 h | PAAG_04974 | adenylosuccinate lyase | 533,51 | 11 | ** | purine nucleotide/nucleoside/nucleobase anabolism |  |
| Pentose phosphate shunt | | | | | | |  |
| 12 h | PAAG_05621 | 6-phosphogluconolactonase | 1007,66 | 4 | ** | Pentose phosphate shunt |  |
| Lipid, fatty acid and isoprenoid metabolism | | | | | | |  |
| 12 h | PAAG_05150 | ATP-citrate synthase subunit 1 | 648,75 | 14 | ** | citrate biosynthethic process |  |
| 12 h | PAAG_05151 | ATP-citrate lyase | 516,36 | 10 | ** | Oxaloacetate and acetyl-CoA from citrate |  |
| 12 h | PAAG_07786 | acetyl-CoA acetyltransferase (acetoacetyl-CoA thiolase) | 1533,08 | 5 | -0.66 | Acetoacetyl-CoA from Acetyl-CoA |  |
| 12 h | PAAG_07279 | farnesyl pyrophosphate synthetase | 420,26 | 8 | ** | isoprenoid metabolism |  |
| 24 h | PAAG_05151 | ATP-citrate-lyase | 561,24 | 16 | ** | acetyl-CoA biosynthethic process |  |
| 24 h | PAAG_05150 | ATP-citrate synthase subunit 1 | 854,51 | 10 | ** | citrate biosynthethic process |  |
| 24 h | PAAG_08553* | fatty acid elongase | 490,75 | 5 | ** | fatty acid metabolism |  |
| 24 h | PAAG_03631 | 12-oxophytodienoate reductase | 893,98 | 10 | ** | oxidative metabolism of polyunsaturated fatty acids. |  |
| 24 h | PAAG_08994 | leukotriene A-4 hydrolase | 64,7 | 1 | ** | lipid and fatty acid metabolism/ leukotriene biosynthetic process |  |
| 24 h | PAAG_03366 | phytanoyl-CoA dioxygenase family protein | 614,13 | 4 | ** | fatty acid alpha-oxidation |  |
| Secondary metabolism | | | | | | |  |
| 12 h | PAAG_01125 | thiazole biosynthesis enzyme | 2716,9 | 5 | -0.65 | Thiamine (vitamin) biosynthesis |  |
| 24 h | PAAG_00851 | 6,7-dimethyl-8-ribityllumazine synthase | 817,86 | 3 | -0.59 | riboflavin biosynthesis |  |
| ENERGY | | | | | | |  |
| Electron transport and membrane-associated energy conservation | | | | | | |  |
| 12 h | PAAG_05576 | ATP synthase gamma chain | 1219,59 | 1 | -1.85 | aerobic respiration |  |
| 12 h | PAAG_04698* | [NADH-ubiquinone oxidoreductase](http://www.ncbi.nlm.nih.gov/blast/Blast.cgi#alnHdr_225561855) | 460,04 | 4 | ** | electron transport and membrane-associated energy conservation |  |
| 12 h | PAAG_07246* | [cytochrome-c oxidase chain VI](http://www.ncbi.nlm.nih.gov/blast/Blast.cgi#alnHdr_225684940) | 778,03 | 5 | ** | respiration |  |
| TCA cycle | | | | | | |  |
| 12 h | PAAG_07843 | aconitate hydratase | 409,8 | 9 | ** | tricarboxylic-acid pathway (citrate cycle, Krebs cycle, TCA cycle) |  |
| 12 h | PAAG_08351 | mitochondrial NADP-specific isocitrate dehydrogenase | 613,79 | 5 | ** | glyoxylate and TCA cycles (oxidative decarboxylation of isocitrate) |  |
| 24 h | PAAG_00856 | isocitrate dehydrogenase subunit 1 | 2008,07 | 8 | ** | TCA cycle |  |
| 24 h | PAAG_00588 | fumarate hydratase | 889,02 | 6 | ** | TCA cycle |  |
| CELL CYCLE and DNA PROCESSING | | | | | | |  |
| 12 h | PAAG_08917 | histone H2a | 922,43 | 4 | -0.58 | chromosome condensation |  |
| 12 h | PAAG_08918 | late histone H2B L4 | 2241,63 | 2 | ** | chromosome condensation |  |
| 12 h | PAAG_01016 | NAD-dependent deacetylase sirtuin-2 | 416,98 | 4 | ** | DNA restriction or modification |  |
| 12 h | PAAG_12254* | telomere-binding alpha subunit central domain-containing protein | 492,92 | 8 | ** | telomere maintenance |  |
| TRANSCRIPTION | | | | | | |  |
| 12 h | PAAG_04726 | pirin | 447,4 | 8 | ** | mRNA synthesis |  |
| 12 h | PAAG_07957* | pre-mRNA-splicing factor srp1 | 443,42 | 3 | ** | splicing |  |
| TRANSLATION | | | | | | |  |
| 12 h | PAAG_00689 | ATP-dependent RNA helicase eIF4A | 2890,58 | 9 | -0.68 | protein biosynthesis |  |
| 12 h | PAAG_09083 | TCTP family protein | 8767,22 | 3 | -0.72 | protein biosynthesis |  |
| 12 h | PAAG_01786 | phenylalanyl-tRNA synthetase beta chain 6 | 432,31 | 7 | ** | aminoacyl-tRNA-synthetases |  |
| 12 h | PAAG_03572 | glutaminyl-tRNA synthetase | 584,11 | 11 | ** | aminoacyl-tRNA-synthetases |  |
| 12 h | PAAG_08702 | seryl-tRNA synthetase | 723,46 | 11 | ** | aminoacyl-tRNA-synthetases |  |
| 12 h | PAAG_07283 | ATP dependent RNA helicase FAL1 | 470 | 10 | ** | translation |  |
| 12 h | PAAG_01425 | eukaryotic translation initiation factor 3 | 611,28 | 3 | ** | translation initiation |  |
| 24 h | PAAG_00376 | eukaryotic translation initiation factor 3 subunit F | 732,19 | 6 | -1,21 | translation |  |
| 24 h | PAAG_00240 | eukaryotic translation initiation factor 5A | 1468,6 | 3 | -1,13 | translation |  |
| 24 h | PAAG_00689 | ATP-dependent RNA helicase eIF4A | 2822,22 | 12 | -1,37 | translation initiation |  |
| 24 h | PAAG_07420* | eukaryotic translation initiation factor 3 subunit K | 575,88 | 4 | ** | translation initiation |  |
| 24 h | PAAG_01425 | eukaryotic translation initiation factor 3 | 806,51 | 6 | ** | translation initiation |  |
| 24 h | PAAG_08817 | translation initiation factor 2 subunit beta | 1232,9 | 4 | ** | translation initiation |  |
| 24 h | PAAG_03572 | glutaminyl-tRNA synthetase | 679,72 | 14 | ** | aminoacyl-tRNA-synthetases/ translation |  |
| 24 h | PAAG_00772* | translation initiation factor eIF3 subunit J | 690,51 | 5 | ** | translation initiation |  |
| PROTEIN FATE | | | | | | |  |
| 12 h | PAAG_04286 | 50S ribosomal protein L1 | 554,55 | 8 | ** | ribosomal proteins |  |
| 12 h | PAAG_08540 | 40S ribosomal protein S25 | 685,39 | 3 | ** | ribosomal proteins |  |
| 12 h | PAAG_08634 | 40S ribosomal protein S12 | 1088,15 | 6 | ** | ribosomal proteins |  |
| 12 h | PAAG_06882 | 40S ribosomal protein S24 | 1990,55 | 7 | ** | ribosome biogenesis |  |
| 24 h | PAAG_05679 | heat shock protein | 3917,5 | 27 | -1.90 | protein folding and stabilization |  |
| 24 h | PAAG_05913* | histone-lysine N-methyltransferase | 29,25 | 1 | -3.51 | histone-modifying enzymes |  |
| 24 h | PAAG_06255 | mitochondrial co-chaperone GrpE | 6159,68 | 6 | -0.68 | protein folding and stabilization |  |
| 24 h | PAAG_06536 | ubiquitin | 6185,71 | 5 | -0.66 | protein degradation |  |
| 24 h | PAAG_06168 | peptidyl-prolyl cis-trans isomerase cypE | 1700,86 | 3 | -0.65 | protein folding and stabilization |  |
| 24 h | PAAG_06287 | carboxypeptidase S1 | 583,52 | 6 | ** | protein/peptide degradation |  |
| 24 h | PAAG_02686 | Hsp90 co-chaperone AHA1 | 634,63 | 6 | ** | protein folding and stabilization |  |
| 24 h | PAAG_01854* | nuclear transport factor 2 | 943,23 | 3 | ** | protein import into nucleus/ protein targeting, sorting and translocation |  |
| 24 h | PAAG_01778 | peptidyl-prolyl cis-trans isomerase H | 1609,19 | 4 | ** | protein folding and stabilization |  |
| 24 h | PAAG_06461 | U-box domain-containing protein | 612,18 | 5 | ** | protein binding/ Ubl conjugation pathway |  |
| TRANSPORT | | | | | | |  |
| 12 h | PAAG_12473* | phospholipid transporting ATPase | 444,91 | 11 | ** | cation transport (H+, Na+, K+, Ca2+ , NH4+, etc.) |  |
| 12 h | PAAG_05036 | transport protein SEC24 | 555,24 | 9 | ** | ER to Golgi transport |  |
| 12 h | PAAG_00326* | metal homeostasis factor ATX1 | 1303,37 | 1 | ** | heavy metal ion transport (Cu+, Fe3+, etc.) |  |
| 12 h | PAAG_03105* | [toxin biosynthesis protein](http://www.ncbi.nlm.nih.gov/blast/Blast.cgi#alnHdr_261205870) | 432,32 | 6 | ** | toxins_protein transport |  |
| CELL RESCUE, DEFENSE and VIRULENCE | | | | | | |  |
| 12 h | PAAG_05679 | heat shock protein | 1859,42 | 19 | -0.62 | stress response/ protein folding and stabilization |  |
| 12 h | PAAG_00871 | 30 kDa heat shock protein | 819,74 | 14 | ** | heat shock response |  |
| 24 h | PAAG_00871 | 30 kDa heat shock protein | 838,76 | 8 | ** | heat shock response |  |
| 24 h | PAAG_03216 | mitochondrial peroxiredoxin PRX1 | 1307,94 | 3 | ** | oxidative stress response |  |
| CELL GROWTH/ MORPHOGENESIS | | | | | | |  |
| 12 h | PAAG_00875 | ARP2/3 actin-organizing complex subunit Sop2 | 768,88 | 5 | ** | cell growth / morphogenesis |  |
| 24 h | PAAG_09083 | TCTP family protein | 7518,55 | 4 | -1.24 | positive regulation of multicellular organism growth |  |
| 24 h | PAAG_07958 | fimbrin | 665,14 | 9 | -0.81 | actin filament binding/ budding, cell polarity and filament formation |  |
| BINDING | | | | | | |  |
| 12 h | PAAG_02567 | EF-hand superfamily Ca2+- modulated protein | 716,62 | 6 | ** | Calcium binding |  |
| 24 h | PAAG_05224* | RNA binding domain-containing protein | 812,99 | 4 | ** | RNA binding |  |
| MISCELLANEOUS | | | | | | |  |
| 12 h | PAAG_02916 | GNAT family N-acetyltransferase | 704,66 | 5 | ** | transferase |  |
| 12 h | PAAG_01302 | phosphorylase family protein | 561,13 | 6 | ** | - |  |
| UNCLASSIFIED | | | | | | | |
| 12 h | PAAG_03701 | BAR domain containing protein | 831,13 | 8 | -2.50 | - |  |
| 12 h | PAAG_00040 | predicted protein | 659,96 | 1 | ** | - |  |
| 12 h | PAAG_00308 | predicted protein | 663,07 | 3 | ** | - |  |
| 12 h | PAAG_00604 | conserved hypothetical protein | 1177,09 | 4 | ** | - |  |
| 12 h | PAAG_01221 | predicted protein | 706,54 | 2 | ** | - |  |
| 12 h | PAAG_02005 | predicted protein | 469,56 | 2 | ** | - |  |
| 12 h | PAAG_02393 | predicted protein | 486,55 | 3 | ** | - |  |
| 12 h | PAAG_02964 | predicted protein | 552,38 | 9 | ** | - |  |
| 12 h | PAAG_03426 | predicted protein | 434,54 | 7 | ** | - |  |
| 12 h | PAAG_04015 | conserved hypothetical protein | 1348,84 | 3 | ** | - |  |
| 12 h | PAAG_04367 | DUF974 domain containing protein | 429,94 | 8 | ** | - |  |
| 12 h | PAAG_06411 | conserved hypothetical protein | 554,68 | 5 | ** | - |  |
| 24 h | PAAG_00331 | DUF431 domain-containing protein | 783,97 | 5 | ** | - |  |
| 24 h | PAAG_05037 | HHE domain containing protein | 791,23 | 2 | ** | - |  |
| 24 h | PAAG_08671 | conserved hypothetical protein | 7,14 | 1 | ** | - |  |
| 24 h | PAAG_04824 | FAS1 domain containing protein | 658,69 | 4 | ** | - |  |
| 24 h | PAAG_03817 | predicted protein | 111,67 | 1 | ** | - |  |
| 24 h | PAAG_09108 | RPEL repeat protein | 952,95 | 1 | ** | - |  |
| 24 h | PAAG_04256 | conserved hypothetical protein | 843,13 | 3 | ** | - |  |

^a^ Identification of differentially regulated proteins from *Paracoccidioides* genome database (<http://www.broadinstitute.org/annotation/genome/paracoccidioides_brasiliensis/MultiHome.html>) using the ProteinLynx Global Server (PLGS) version 3.0 (Waters Corporation. Manchester. UK);

^b^ Proteins annotation from *Paracoccidioides* genome database or by homology (indicated using “*”) from NCBI database (<http://www.ncbi.nlm.nih.gov/>);

^c^  Average of protein score and matched peptides for each protein obtained from MS data using the ProteinLynx Global Server (PLGS);

^d^ Protein expression profiles in log2 (fold change) obtained from ProteinLynx Global Server (PLGS) analysis normalized with internal standard.

^e^ Biological process of differentially expressed proteins from MIPS

(<http://pedant.helmholtz-muenchen.de/pedant3htmlview/pedant3view?Method=analysis&Db=p3_r48325_Par_brasi_Pb01> ) and Uniprot database (<http://www.uniprot.org/>).

**: identified only in normoxia condition.
