# Supplementary material for: Characterization of the Paracoccidioides Hypoxia Response Reveals New Insights into Pathogenesis Mechanisms of This Important Human Pathogenic Fungus
Source: PLoS Negl Trop Dis. 2015 Dec 10;9(12):e0004282. doi: 10.1371/journal.pntd.0004282 (PMC4686304; doi:10.1371/journal.pntd.0004282)
Supplement: S3 Table — (DOCX) [file pntd.0004282.s007.docx]

**S3 Table. Oligonucleotides used in this study.**

| Gene | Procedure | Forward primer | Reverse primer |
| --- | --- | --- | --- |
| *PbsrbA* | qRT-PCR (*Paracoccidioides*) – Figure 6, 9 and S3 | CTA GTG AGG GTC GAA CAA | CTG GCT CAA ACT TCC ATA C |
| *tubulin alpha-1 chain* | qRT-PCR (*Paracoccidioides*) – Figure 4, 6 and 9 | ACA GTG CTT GGG AAC TAT ACC | GGG ACA TAT TTG CCA CTG CC |
| *erg 3* | qRT-PCR (*Paracoccidioides*) – Figure 4 | GGA GAA TAT GTA TAC CAG CCC | ATC CAA GTG ATG AGA TAC AGA G |
| *gpdA + PbsrbA* | Conventional PCR – Figure S2  Primer A (forward) and B (reverse) | GCA CTA TTG ATC ATC CGA TAG C | CTA TCA TAG TGG TGT CTG CAG T |
| *gpdA* | Conventional PCR – Figure S2  Primer A (forward) and C (reverse) | GCA CTA TTG ATC ATC CGA TAG C | TGT GAT GTC TGC TCA AGC GGG GT |
| *AfsrbA* | qRT-PCR (*Aspergillus fumigatus*) – Figure S3 | CAG CCA GCA AAC TCA ACA AAG | GCG GTA TTC TCT TCC TCC AATC |
| *tefA* | qRT-PCR (*Aspergillus fumigatus*) – Figure S3 | GTG ACT CCA AGA ACG ATC CC | AGA ACT TGC AAG CAA TGT GG |
